# EADD-YOLO: An efficient and accurate disease detector for apple leaf using improved lightweight YOLOv5

**DOI:** 10.3389/fpls.2023.1120724

**Published:** 2023-02-23

**Authors:** Shisong Zhu, Wanli Ma, Jianlong Wang, Meijuan Yang, Yongmao Wang, Chunyang Wang

**Affiliations:** ^1^ School of Computer Science and Technology, Henan Polytechnic University, Jiaozuo, China; ^2^ School of Artificial Intelligence, Optics and Electronics (iOPEN), Northwestern Polytechnical University, Xi’an, China

**Keywords:** apple leaf, disease detection, depthwise convolution, coordinate attention, SIoU loss

## Abstract

**Introduction:**

Current detection methods for apple leaf diseases still suffer some challenges, such as the high number of parameters, low detection speed and poor detection performance for small dense spots, which limit the practical applications in agriculture. Therefore, an efficient and accurate model for apple leaf disease detection based on YOLOv5 is proposed and named EADD-YOLO.

**Methods:**

In the EADD-YOLO, the lightweight shufflenet inverted residual module is utilized to reconstruct the backbone network, and an efficient feature learning module designed through depthwise convolution is proposed and introduced to the neck network. The aim is to reduce the number of parameters and floating point of operations (FLOPs) during feature extraction and feature fusion, thus increasing the operational efficiency of the network with less impact on detection performance. In addition, the coordinate attention module is embedded into the critical locations of the network to select the critical spot information and suppress useless information, which is to enhance the detection accuracy of diseases with various sizes from different scenes. Furthermore, the SIoU loss replaces CIoU loss as the bounding box regression loss function to improve the accuracy of prediction box localization.

**Results:**

The experimental results indicate that the proposed method can achieve the detection performance of 95.5% on the mean average precision and a speed of 625 frames per second (FPS) on the apple leaf disease dataset (ALDD). Compared to the latest research method on the ALDD, the detection accuracy and speed of the proposed method were improved by 12.3% and 596 FPS, respectively. In addition, the parameter quantity and FLOPs of the proposed method were much less than other relevant popular algorithms.

**Discussion:**

In summary, the proposed method not only has a satisfactory detection effect, but also has fewer parameters and high calculation efficiency compared with the existing approaches. Therefore, the proposed method provides a high-performance solution for the early diagnosis of apple leaf disease and can be applied in agricultural robots. The code repository is open-sourced at https://github.com/AWANWY/EADD-YOLO.

## 1 Introduction

Apple has rich nutritional and medicinal values and is cultivated worldwide. In addition, apples are used in China as a critical crop to promote poverty alleviation and prosperity among farmers because of their high comparative efficiency. In 2019, China produced more than 41 million tons of apples, accounting for 54.07% of the global total, making it the largest apple producer in the world (HU et al., 2022). Due to environmental impact and bacterial infection, various leaf diseases will occur during apple growth. If these diseases cannot be identified and prevented in time, they can easily cause a sharp decline in apple yield and quality, resulting in substantial economic losses (Bansal et al., 2021; Yogeshwari and Thailambal, 2021). Therefore, the timely diagnosis and treatment of foliar diseases are of great relevance to the sustainable and healthy development of the apple industry.

Traditionally, growers identify the type of disease spots by visual inspection. However, the method of manual discrimination has high work intensity and a considerable risk of misjudgment (Dutot et al., 2013; Lin et al., 2022). With the development of computer technology, machine learning-based methods have been widely applied to recognize disease leaves (Guru et al., 2011; Majumdar et al., 2015; Xie et al., 2016; Pantazi et al., 2019; Hamdani et al., 2021; Ngugi et al., 2021; Pallathadka et al., 2022). For example, Pallathadka et al. (2022) performed histogram equalization on the image, applied the principal component analysis algorithm to extract features, and then utilized the support vector machine to classify leaf diseases. Majumdar et al. (2015) extracted the characteristics of wheat diseases by Fuzzy C-Means and then identified disease spots employing the artificial neural network. Guru et al. (2011) obtained the diseased area features by contrast stretching transformation with an adjustable parameter and morphological operation and classified seedling diseases such as anthracnose and frog-eye spots on tobacco using the product-based neural network. However, the image preprocessing and feature extraction of these machine learning-based methods require much computing work and rely heavily on expert experience (Xie et al., 2016; Ngugi et al., 2021), which limits the migration ability and practicability of these methods.

Recently, deep learning-based methods have made significant breakthroughs in crop leaf disease identification because of their excellent feature extraction and model migration ability. Liu et al. (2022) and Bhujel et al. (2022) investigated lightweight disease classification networks with good accuracy based on the cucumber and tomato leaf disease datasets, respectively. Moreover, Zhu et al. (2022) have attempted to deploy the lightweight apple early leaf disease recognition model on the mobile end. With the emergence of target detection models, such as Faster-RCNN (Ren et al., 2015), SSD (Liu et al., 2016), and YOLO series (Redmon et al., 2016; Redmon and Farhadi, 2017; Redmon and Farhadi, 2018; Bochkovskiy et al., 2020; Wang et al., 2022a), they can provide accurate location information alongside identifying disease spots, thus attracting an increasing number of researchers to employ them in agriculture for precise classification and localization of diseased areas on crop leaves (Shrestha et al., 2020; Pan et al., 2022). However, most disease spot detection methods have a large model size, which is not convenient for deployment on mobile devices, making them difficult to meet practical applications in agriculture (Jiang et al., 2019; Ozguven and Adem, 2019; Temniranrat et al., 2021). For instance, Jiang et al. (2019) devised the INAR-SSD to detect five common apple leaf diseases. The method improves the detection performance of the SSD network for various disease spots through the inception module and the rainbow connections. Due to the stacking of the extensive inception modules and complex skip connections, INAR-SSD has a high parameter quantity and is not suitable for the mobile end. In recent years, more attention has been focused on reducing the complexity of the model to enhance the practicality of the network (Atila et al., 2021; Wu et al., 2021; Naik et al., 2022). Nalepa et al. (2020) exploited quantization-aware training with additional fine-tuning to save memory and energy-frugal and make deep convolutional neural networks easier to deploy on resource-constrained mobile or hardware devices. In addition, many scholars working in smart agriculture have studied the structural optimization of deep convolutional neural networks and applied them to the task of crop leaf disease detection. For example, Sun et al. (2021) utilized the mobile end applenet (MEAN) block with the group convolutions in the backbone network of MEAN-SSD to increase the speed of detecting early apple leaf diseases. Although this approach has made some attempts to optimize the network structure, its high computational cost and low detection accuracy on small spots caused by the heavy use of the group convolution still limit applications in practical scenes. Because of the efficiency, flexibility, and good generalization performance of YOLO networks, YOLO-based disease detection algorithms have become a research hotspot (Khan et al., 2022). For instance, Liu and Wang (2020) applied the lightweight classification network MobileNetv2 as the feature extractor in the MobileNetv2-YOLOv3 to improve operational efficiency. However, the large number of convolution and bottleneck modules in the neck network results in the model still having many parameters. Wang et al. (2022b) introduced the BiFPN structure to alleviate the low detection accuracy of the modified YOLOv5 but inevitably raised computational costs, which led to higher complexity. Although these crop leaf disease detection methods have attempted to optimize the structure of the network to improve computational efficiency, they do not make efficient measures to cope with the reduction in precision, resulting in low detection performance or only a slight decrease in model complexity. To address these problems, an efficient and accurate spot detection model EADD-YOLO is proposed in this study to compress the model size while maintaining the precision required for practical applications. The superior performance of the proposed method has been demonstrated on apple leaf disease images. The main contributions are as follows:

- The backbone network of EADD-YOLO consists of several lightweight shufflenet inverted residual modules to reduce the parameter quantity and FLOPs, making feature extraction more efficient.
- The lightweight DWC3 module designed employing depthwise convolution is proposed to replace the original C3 module in the neck network, which is to further decrease the model complexity and enhance the detection speed of the network while maintaining the expression ability of features.
- The coordinate attention module is embedded at critical locations in the network to highlight crucial spot information, which can improve detection accuracy without significantly increasing computational costs.
- SIoU loss with introducing an angle cost is utilized as the regression loss function of the bounding box to alleviate the low regression accuracy of the prediction boxes during training.

The rest of the paper is arranged as follows: Section 2 shows the details of the dataset. In addition, the principle of YOLOv5 and the design of the proposed EADD-YOLO are introduced in Section 2. Then, Section 3 presents and analyzes the experimental results. Next, the comparison and discussion of the proposed method with the relevant popular methods are demonstrated in Section 4. Finally, Section 5 summarizes the work of this study and prospects for the future research direction.

## 2 Materials and methods

### 2.1 Dataset

**Figure 1:**
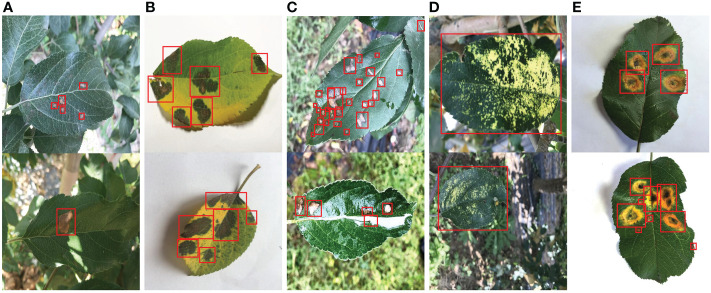
Representative images of five types of apple leaf disease spots. **(A)** Alternaria blotch. **(B)** Brown spot. **(C)** Grey spot. **(D)** Mosaic. **(E)** Rust. The red rectangle indicates the annotation box (ground truth).

The apple leaf disease dataset (ALDD) used in this study was from Northwest A&F University. The dataset adopts manual photography to obtain the disease images of apple leaves in indoor and outdoor scenes. The images of outdoor scenes were collected on sunny days, cloudy days, rainy days, and other weather conditions. Five common apple leaf diseases were covered in the dataset: Alternaria blotch (caused by Alternaria alternata f. sp mali), brown spot (caused by Marssoninacoronaria), grey spot (caused by Phyllosticta pirina Sacc. and Coryneum foliicolum), mosaic (caused by Papayaringspot virus), and rust (caused by Pucciniaceae glue rust). The other four disease images include indoor and outdoor scenes except for the brown spot. In addition, the images in the dataset were enhanced by folding, rotation, brightness, and contrast changes. Finally, there are 26377 images in the dataset, with a size of 512 × 512. **Figure 1** shows representative images of five types of apple leaf diseases, and the specific number of images for each category is provided in **Table 1**.

**Table 1:** The number of images of five types of apple leaf disease spots.

Disease	Number		
	Indoor	Outdoor	Total
Alternaria blotch	1755	3588	5343
Brown spot	5655	0	5655
Grey spot	2288	2522	4875
Mosaic	2782	2093	4810
Rust	5486	208	5694
Total			26377

In this work, LabelImg is applied to mark the location and category of disease spots in all images in ALDD. Each annotated original image generates a corresponding annotation file in XML format. It contains information such as the file name of the corresponding original image, the image size, the disease type, and the location of the annotation box for each spot. The location of the annotation boxes for several representative images is presented in **Figure 1**, and the corresponding annotation files for these images are provided in the supplementary material. The annotation results were repeatedly verified and corrected to avoid the impact of manual limitations on the experimental results.

As illustrated in **Figure 1**, there are many challenges in apple leaf spot detection: 1) the shape, size and other features of different types of disease spots are diverse, which increases the difficulty of feature extraction. 2) Most spots are small and densely distributed, making localization more difficult. 3) Light spots and raindrops in images from outdoor conditions can interfere with disease identification.

### 2.2 Design for EADD-YOLO

**Figure 2:**
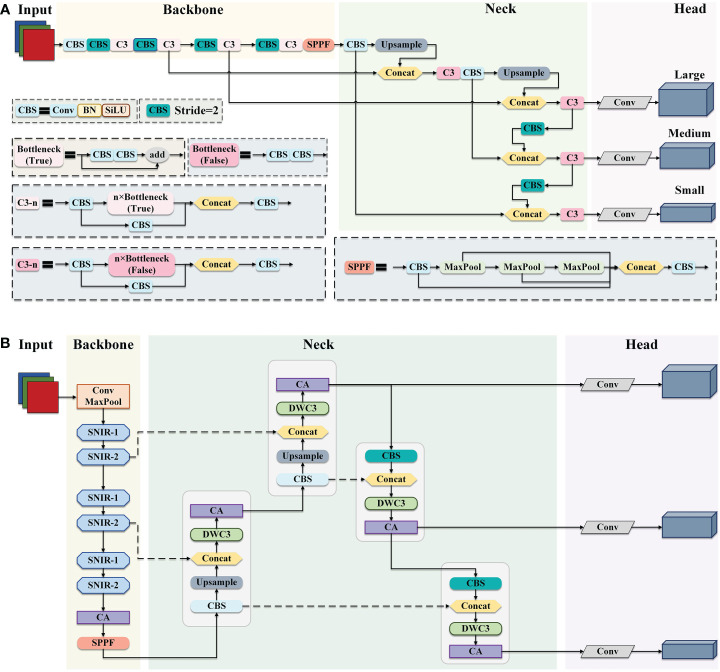
The framework of YOLOv5 and EADD-YOLO. **(A)** The structure of YOLOv5. **(B)** The architecture of the proposed EADD-YOLO.

To reduce the model complexity and improve the detection efficiency while maintaining the accuracy of apple leaf disease identification, an efficient and accurate detection network EADD-YOLO based on YOLOv5, is proposed in this study. **Figures 2A, B** display the exact structure of the YOLOv5 and the proposed EADD-YOLO, respectively.

As presented in **Figure 2**, YOLOv5 and the proposed EADD-YOLO contain four main components: the input layer, the backbone network, the neck network, and the prediction head. From **Figure 2A**, CBS is the basic unit in YOLOv5,consisting of ordinary convolution and batch normalization (BN) and the activation function SiLU. The backbone network of YOLOv5 is composed of a large number of CBS modules and C3 modules stacked from CBS modules to reduce feature dimensionality and extract semantic information. The fast spatial pyramidal pooling (SPPF) module obtains rich multi-scale features through max-pooling with three different kernel sizes and transports them to the neck network. In addition to the CBS and C3 and upsampling modules, concat is utilized in the neck network to aggregate deeper and shallower features, thus reducing information loss. The prediction layer contains three prediction heads at different scales, which can output detection results at different scales.

Due to the large number of CBS and C3 modules in the backbone and neck networks, the original YOLOv5 is challenging to deploy on resource-constrained mobile devices, which limits its application in agriculture. Therefore, a simple and efficient EADD-YOLO is proposed in this work to detect apple leaf disease, as illustrated in **Figure 2B**. The main improvements are as follows: 1) several efficient shufflenet inverted residual (SNIR) modules (light blue) are adopted to replace the stacked CBS and C3 modules to devise the backbone network of the proposed EADD-YOLO. The aim is to reduce the number of parameters and FLOPs generated in the feature extraction process, thus compressing the model size. 2) Moreover, the novel DWC3 module (light green) is designed to replace the original C3 module in the neck network to enhance the efficiency of the feature fusion. 3) In addition, the lightweight coordinate attention (CA) module (light purple) is embedded in the backbone and neck networks to improve the precision of the compressed model on various diseases by highlighting the critical information of spots while introducing less computational cost.

The structure of the SNIR module is given in subsection 2.2.1. The implementation process of the DWC3 module is shown in subsection 2.2.2. In addition, the principle of the CA module is described in detail in subsection 2.2.3.

#### 2.2.1 Lightweight backbone network establishment

The original backbone network of the YOLOv5 contains many CBS and C3 modules, which are mainly composed of ordinary convolution and residual connections with high parameter quantities and FLOPs. To compress the model size and improve its portability with less loss of detection accuracy, the efficient SNIR module is utilized to design the lightweight backbone network of EADD-YOLO.

**Figure 3:**
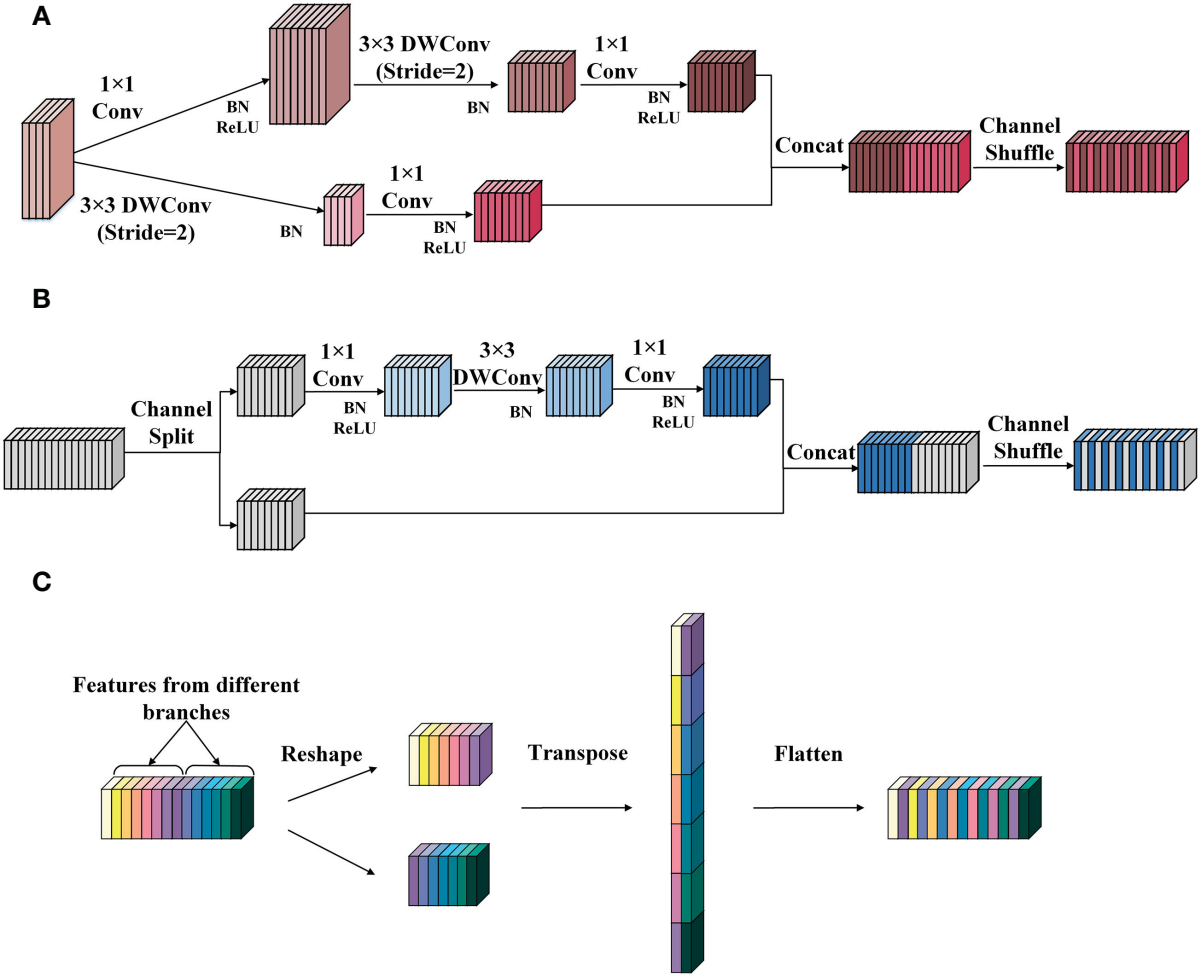
The structure of the SNIR module. **(A)** SNIR-1. **(B)** SNIR-2. **(C)** The channel shuffle operation. Conv represents the ordinary convolution, and the DWConv denotes the depthwise convolution in the depthwise separable convolution. The channel shuffle consists of three steps: (1) reshape: reshape the input channel from one dimension into two dimensions. One is the number of convolution groups, and the other is the number of channels contained in each convolution group. (2) Transpose: swap the two dimensions. (3) Flatten: flatten the transposed channel back as the input of the next layer.

The SNIR module comes from the basic block of the lightweight classification network ShuffleNetv2 (Ma et al., 2018). It can reduce the number of parameters and calculations in the feature extraction process and achieve a good balance between speed and accuracy. According to the different functions performed, the SNIR module can be divided into the SNIR-1 and the SNIR-2. The former halves the height and width of the input feature map and expands the number of channels to four times the original, and the latter only extracts features without changing the size and the number of channels of the input feature map. The working principles of SNIR-1 and SNIR-2 are demonstrated in **Figures 3A, B**, respectively.

As shown in **Figure 3A**, SNIR-1 first transports the input feature map into two branches. Both branches apply depthwise convolution (DWConv) with a step size of 2 to extract semantic information and reduce the height and width of the feature map. Then, the outputs of the two branches are joined to obtain the feature map with the number of channels quadrupled and the size halved than the input. Finally, the channel shuffle operation is performed to integrate the features.

**Figure 3B** illustrates the implementation flow of the SNIR-2. SNIR-2 first divides the input feature map equally by the number of channels and transports them into two separate branches. Then, different operations are performed on the feature map entering the two branches. Specifically, the feature map of one branch remains unchanged (identity mapping). In contrast, the feature map of the other branch will undergo three convolutions (including DWConv) with a step size of 1 to recode the features. Next, the output of the two branches is concatenated. Finally, the features are fused through channel shuffle operation. The principle of channel shuffle operation is displayed in **Figure 3C**.

As shown in **Figure 3C**, the channel shuffle operation breaks up and regroups the channels of the feature map by reshaping and transposing them, which can quickly complete the fusion of information between channels without increasing the computational cost, freeing up the channel information constraints due to convolution operations.

In addition, the channel splitting and branching operations in the SNIR-2 module significantly reduce the computational cost, thus allowing for more efficient feature encoding and transmission. Suppose that the size of the feature map input to the SNIR-2 module is *h* × *w* and the number of channels is *c*. Then, the number of parameters generated by the two branches of the SNIR-2 module is 1^2^·*c/*2·*c*/2+ 3^2^·*c*/2+1^2^ and 0, respectively. In comparison, the number of parameters incurred by simultaneously manipulating all channels of the input feature map is 1^2^·*c*·*c +* 3^2^·*c* + 1^2^·*c*·*c*. As illustrated in equation (1), the parameter quantities generated by applying the channel splitting operation is about 1/4 of that generated by not using this operation. Moreover, the FLOPs generated employing channel splitting are about 1/4 of those caused by the conventional operation when assuming that the feature map does not change in size or the number of channels during every convolution operation, as shown in equation (2). This demonstrates the superiority of the SNIR-2 module in terms of computational cost.


(1) $$r_{Params}=\frac{1^{2}\cdot \frac{c}{2}\cdot \frac{c}{2}+3^{2}\cdot \frac{c}{2}+1^{2}\cdot \frac{c}{2}\cdot \frac{c}{2}+0}{1^{2}\cdot c\cdot c+3^{2}\cdot c+1^{2}\cdot c\cdot c}=\frac{\frac{1}{2}\cdot c\cdot c+\frac{9}{2}.c}{2\cdot c\cdot c+9\cdot c}\approx \frac{1}{4}$$

(2) $$r_{FLOPs}=\frac{1^{2}\cdot \frac{c}{2}\cdot h\cdot w\cdot \frac{c}{2}+3^{2}\cdot \frac{c}{2}\cdot h\cdot w+1^{2}\cdot \frac{c}{2}\cdot h\cdot w\cdot \frac{c}{2}+0}{1^{2}\cdot c\cdot h\cdot w\cdot c+3^{2}\cdot c\cdot h\cdot w+1^{2}\cdot c\cdot h\cdot w\cdot c}=\frac{\frac{1}{2}\cdot c\cdot c+\frac{9}{2}\cdot c}{2\cdot c\cdot c+9\cdot c}\approx \frac{1}{4}$$

In summary, the SNIR module has three advantages compared to the CBS and C3 modules: 1) channel splitting and branching operations in the SNIR module allow for more efficient model training. 2) The channel shuffle operation enables channels to be quickly disrupted and reallocated, exchanging information and enriching features, which can effectively enhance feature representation, thus making the SNIR module balance computational efficiency and detection accuracy. 3) The DWConv with a lower computational cost is introduced to replace ordinary convolution in the SNIR module, thus reducing the number of parameters while maintaining the performance of the network. The superiority of DWConv compared to ordinary convolution will be illustrated when the novel DWC3 module is proposed in subsection 2.2.2.

Therefore, the SNIR-1 and the SNIR-2 modules are alternately connected in series to design the lightweight backbone network of the proposed EADD-YOLO to accomplish fast feature extraction and transmission, as presented in **Figure 2**. Compared to the original backbone network consisting of ordinary convolution, the designed lightweight backbone not only significantly reduces the parameter quantity and computational costs, but also has little impact on the detection accuracy.

#### 2.2.2 Efficient neck network design

To further compress the model size, the novel DWC3 module employing DWConv is proposed and embedded in the neck network to replace the original C3 module, thus improving the efficiency of the model in the feature fusion stage.

**Figure 4:**
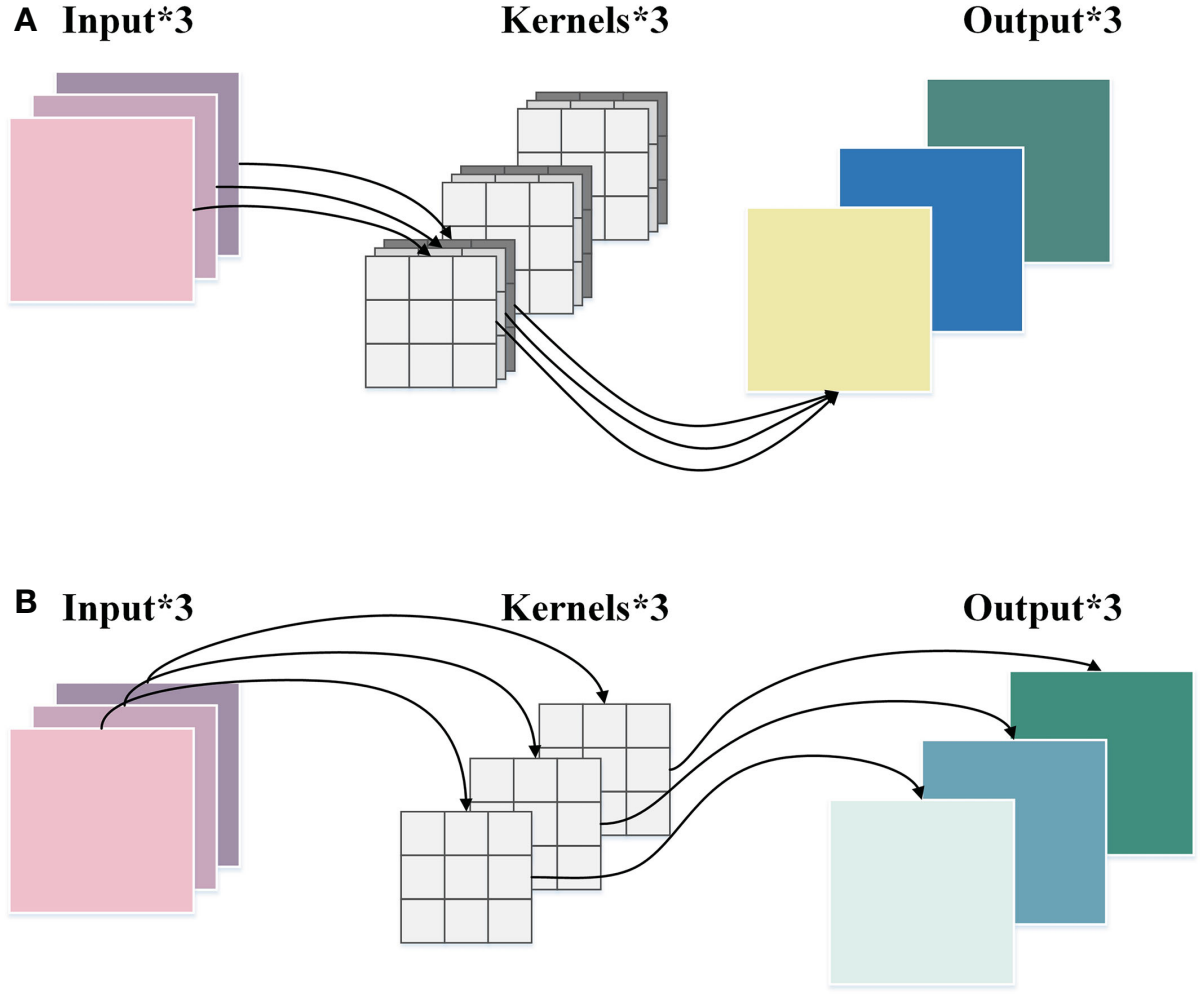
The comparison between the ordinary convolution and the DWConv. **(A)** The ordinary convolution. **(B)** DWConv.

DWConv is a convolution strategy in depthwise separable convolution (Chollet, 2017). It has lower parameters and computational costs than ordinary convolution, which can enhance the efficiency of feature extraction and maintain accuracy. The implementation process of ordinary convolution and DWConv is demonstrated in **Figure 4**.

As displayed in **Figure 4A**, in the ordinary convolution, each kernel must operate on each channel of the input picture at the same time. Then the convolution results of each channel are weighted sum to generate a feature map. While in the DWConv, as shown in **Figure 4B**, each channel of the input image is performed by only one kernel, which then generates a corresponding feature map. The height and width of the input feature map are denoted by *h* and *w*, respectively. The number of channels is *c*, and *k × k* represents the size of the convolution kernel. Therefore, the ratios of parameter quantity and FLOPs between the DWConv and the ordinary convolution are calculated as follows:


(3) $$r_{Params}=\frac{Params_{DWConv}}{Params_{OrdinaryConvlution}}=\frac{k^{2}\cdot c}{k^{2}\cdot c\cdot c'}=\frac{1}{c'}$$

(4) $$r_{FLOPs}=\frac{FLOPs_{DWConv}}{FLOPs_{OrdinaryConvlution}}=\frac{k^{2}\cdot c\cdot h'\cdot w'}{k^{2}\cdot c\cdot h'\cdot w'\cdot c'}=\frac{1}{c'}$$

where *h*’ and *w*’ denote the height and width of the output feature map, respectively, and *c*’ indicates the number of channels of the output feature map. From Equation (3) and Equation (4), it can be concluded that the parameter quantity and FLOPs of DWConv are only 1/*c*’ of that of ordinary convolution, which shows the superiority of DWConv in computational efficiency.

**Figure 5:**
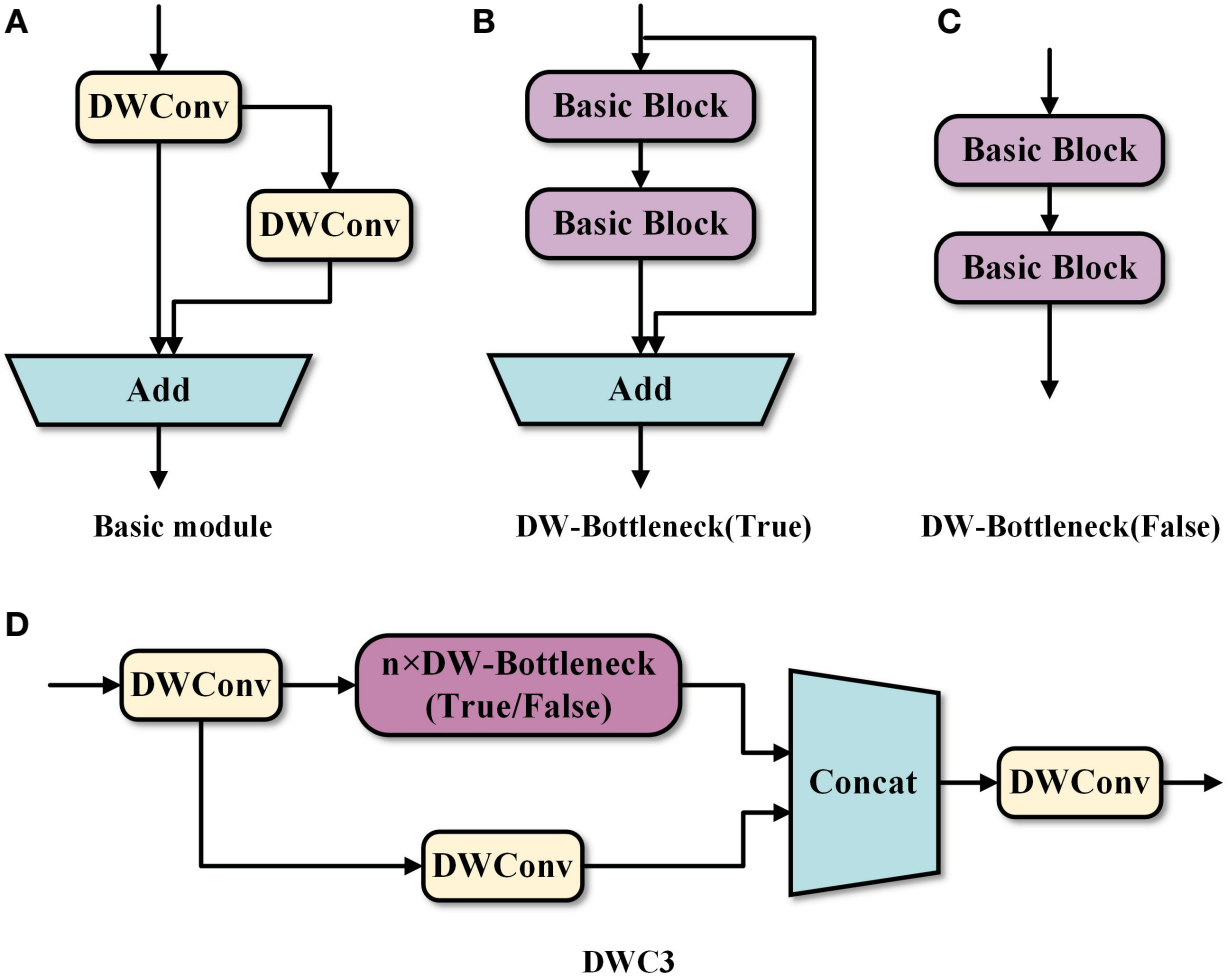
**(A)** Basic module. **(B)** DW-Bottleneck(True). **(C)** DW-Bottleneck(False). **(D)** DWC3.

Therefore, we proposed an efficient basic block by applying DWConv, as illustrated in **Figure 5A**. In the basic block, the input feature map first goes through DWConv to extract features and is then transported into two branches. One of the branches performs DWConv on the feature map to obtain detailed information. The other branch does not perform any operations. Finally, the results of the two branches are summed for feature aggregation. Compared to the CBS module including only one ordinary convolution, the basic block with the simpler DWConv has less computational cost in extracting features. In addition, the branched convolution introduces more detailed information to ensure the effectiveness of feature extraction in the basic block.

Due to its simplicity and effectiveness, the proposed basic block is utilized to replace the ordinary convolution in the original bottleneck block to form a lightweight DW-Bottleneck, as shown in **Figures 5B, C**. Then, the novel DW-Bottleneck is introduced in the original C3 module, and the CBS module is replaced by DWConv, thus devising the efficient DWC3 module, as illustrated in **Figure 5D**. The proposed DWC3 module is embedded into the neck network to enhance the efficiency of the feature fusion. In this way, the computational cost of the neck network can be decreased while maintaining the expressiveness of the features, thus reducing the impact on detection accuracy.

#### 2.2.3 Coordinate attention module

The lightweight improvement of the backbone and neck network can remarkably reduce the number of parameters and FLOPs of the model and increase the detection speed of the network while inevitably causing some loss in detection accuracy. So, it is necessary to optimize the network further to enhance the detection performance of the model for different diseases. Therefore, the CA modules are introduced in the critical positions of the network to increase the sensitivity of the model to the characteristics of disease spots, thus enhancing the ability of the network to identify and locate small spots without significantly affecting computational efficiency.

**Figure 6:**
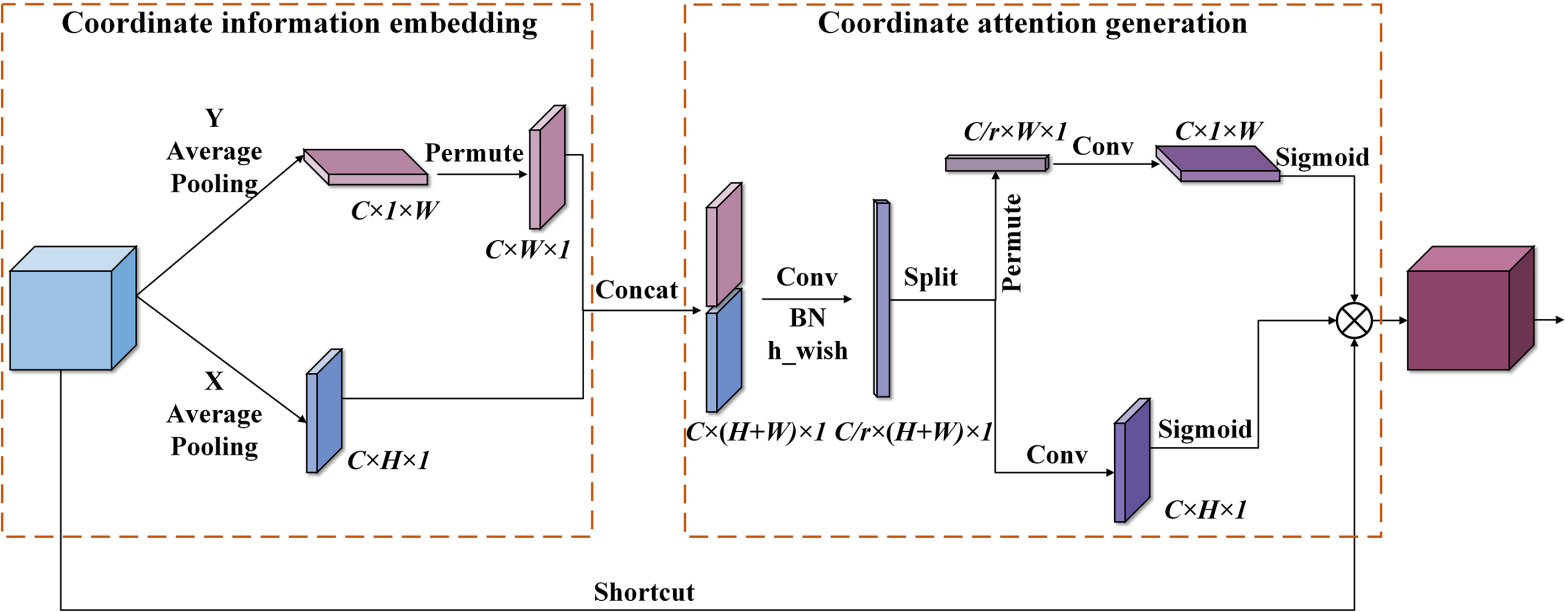
The implementation process of the coordinate attention module. Conv indicates convolution operation, Concat represents a fusion of feature maps by channel stitching, and permute denotes matrix transposition operation.

The CA applies a flexible and lightweight coordinate attention mechanism, which can improve the efficiency and accuracy of image information processing (Hou et al., 2021). By embedding the location information into the channel attention, the CA module can make the network obtain the information of a larger area and avoid incurring a high computational cost. The implementation flowchart of the CA module is displayed in **Figure 6**.

As shown in **Figure 6**, the coordinate attention mechanism can be summarized into coordinate information embedding (CIE) and coordinate attention generation (CAG). In the stage of CIE, features are gathered. All channels of the input feature map are operated by the average pooling along the horizontal and vertical coordinates to obtain features with accurate location information. The dimensions are *C* × *H* × 1 and *C* × 1 × *W*, respectively. In the stage of CAG, the two feature maps with accurate coding information are concatenated. Next, 1 × 1 Conv is utilized to compress its channel dimension from the *C* to the *C*/*r* dimensions. Then, the h_wish function is used to perform nonlinear activation, thus obtaining the intermediate feature representing the encoded information. Subsequently, the intermediate feature is decomposed along the spatial dimension into a vertical attention tensor (*C/r* × 1 × *W*) and level attention tensor (*C/r* × *H* × 1). After that, two sets of 1 × 1 Conv are applied to increase the channel dimension of the obtained two tensors from *C*/*r* to *C*. Then, the sigmoid function is utilized for nonlinear activation to generate attention weight. Finally, the obtained attention weights in the two directions are multiplied by the input feature map to complete the application of coordinate attention.

The embedded location information allows the network access to a more extensive information area, thus improving prediction accuracy. In addition, the implementation of the CA module only uses convolution operations with the kernel size of 1 × 1, and a few matrix transposition operations except for two average pooling operations, leading to less computational cost. So, it has a low impact on the operational efficiency of the network.

Therefore, the lightweight CA module is embedded in the backbone network and the information intersection of the neck network of the EADD-YOLO to increase the identification ability of the model for diseases with various characteristics. The improvement can benefit the network in selecting the critical information for the disease spot detection task, and improve the effectiveness and accuracy of the neck network in processing feature information, thus alleviating the loss of accuracy due to model compression without incurring high computational costs.

### 2.3 Loss function improvement

The original YOLOv5 utilizes CIoU loss (Zheng et al., 2021) to calculate the difference between the prediction and target boxes, since it takes the aspect ratio of the bounding boxes into the loss function, effectively improving the regression accuracy. However, ignoring the problem of directional mismatch between the target box and the prediction box, CIoU loss is prone to the phenomenon that the prediction box wanders around the target box during the training process, which results in low accuracy of the network.

Therefore, SIoU loss (Gevorgyan, 2022) is employed as a regression loss function of the detection box in this work. It introduces an angle cost that considers the relationship between the orientation of the predicted box and the true box, thus enhancing the localization accuracy of the prediction box.

SIoU loss consists of IoU cost, shape cost, and distance cost defined by introducing an angle cost. It is calculated as:


(5) $$Loss_{SIoU}=1-IoU+\frac{\text{Ω}+\text{Δ}}{2}$$

where (1−*IoU*) refers to the IoU cost, Ω indicates the shape cost, and Δ represents the distance cost with an angle cost introduced. The definition and calculation formulae for IoU, the shape cost, the distance cost and the angle cost are explained in turn in the following text.

IoU refers to the intersection over union ratio between the true box and the predicted box, which describes their coincidence degree. It is defined as:


(6) $$IoU=\frac{B\cap B^{gt}}{B\cup B^{gt}}$$

where *B* and *B^gt^* represent the prediction box area and the real box area, respectively.

The shape cost considers the aspect ratio between the target box and the predicted box to make their shapes more similar. It is calculated as:


(7) $$\text{Ω}=\sum_{t=w,h}{\left(1-e^{-\omega_{t}}\right)}^{\theta }$$

(8) $$\omega_{w}=\frac{\left|w-w^{gt}\right|}{max\left(w,w^{gt}\right)},\omega_{h}=\frac{\left|h-h^{gt}\right|}{max\left(h,h^{gt}\right)}$$

where (*w*,*h*) and (*w^gt^*, *h^gt^*) denote the width and height of the predicted box and the true box, respectively. *θ* controls the degree of attention of the loss function to shape cost. To avoid reducing the movement of the prediction box by paying too much attention to the shape cost, the value of *θ* is set to 4 in this work within the suggested range of values (*θ* ∈ [2,6] according to Gevorgyan (2022)).

The distance cost describes the distance between the central points of the prediction and the target boxes. It is defined as:


(9) $$\text{Δ}=\sum_{t=x,y}\left(1-e^{-\gamma \rho_{t}}\right)$$

where,


(10) $$\rho_{x}={\left(\frac{b_{c_{x}}^{gt}-b_{c_{x}}}{d_{w}}\right)}^{2},\rho_{y}={\left(\frac{b_{c_{y}}^{gt}-b_{c_{y}}}{d_{h}}\right)}^{2},\gamma =2-\text{Λ}$$

Where $\left(b_{c_{x}}^{gt},b_{c_{y}}^{gt}\right)$ denotes the coordinate of the central point of the target box, while (*b*_*c*_*x*__,*b*_*c*_*y*__) refers to the coordinate of the center point of the predicted box. *d_w_* and *d_h_* represent the width and height of the minimum closure bounding box of the prediction box and the target box, respectively. Δ indicates the angle cost that considers the magnitude of the angle between the central points of the true and the predicted boxes. The angle cost is calculated as:


(11) $$\text{Λ}=1-2*\overset{2}{\sin}(\arcsin\;(\sin\;(\alpha ))-\frac{\pi }{4})$$

(12) $$\sin\;(\alpha )=\frac{c_{h}}{\sigma }$$

where *α* ∈ [0,π/2] means the angle between the horizontal line and the line connecting the central points of the prediction box and the real box. *c_h_* refers to the height difference between the central points of the target box and the predicted box. *σ* denotes the distance between the central points of the target box and the prediction box. Their calculations are as follows:


(13) $$\sigma =\sqrt{{\left(b_{c_{x}}^{gt},b_{c_{x}}\right)}^{2}+{\left(b_{c_{y}}^{gt},b_{c_{y}}\right)}^{2}}$$

(14) $$c_{h}=max\left(b_{c_{y}}^{gt},b_{c_{y}}\right)-min\left(b_{c_{y}}^{gt},b_{c_{y}}\right)$$

From Equation (11), it can be found that when *α* = 0 or *π*/2, the value of the angle cost is 0. It means that the prediction box is on the same horizontal (or vertical) line as the real box, and no further optimization is needed. When *α∈* (0,*π*/4), it indicates that the prediction box is nearer to the horizontal line where the real box is located. At this time, the angle cost increases as *α* increases. So, the angle cost prefers to optimize *α* so that the prediction box is closer to the horizontal on which the true box is located. Conversely, when *α ∈* (*π/*4,0), the angle cost decreases as *α* increases. Thus the angle cost tends to optimize the complementary angle of *α* so that the prediction box is closer to the vertical line on which the real box lies. In this way, the prediction box is quickly brought to the horizontal or vertical line where the target box is located, which can reduce the freedom of the prediction box to wander, thus enhancing the location accuracy.

The angle cost is introduced into the distance cost, as illustrated in Equation (9). From Equations (9) and (11), it is evident that when *α* approaches 0, the contribution of distance loss decreases as the angle cost reduces. In contrast, the closer *α* is to *π*/4, the greater the distance cost, and then the overall loss becomes more significant. In other words, the problem becomes more complex as the angle between the prediction box and the real box increases, and the accuracy will be negatively affected. It demonstrates the necessity and importance of introducing and optimizing angle cost.

Compared with CIoU loss, SIoU loss considers the direction matching between the predicted and target boxes. The angle cost can quickly pull the prediction box onto the axis closest to the target box by minimizing the angle between them, which can reduce the phenomenon of the predicted box wandering around the target box, thus significantly improving the positioning accuracy of the prediction box. Therefore, SIoU loss is introduced in this study to replace CIoU loss as the localization loss of the bounding box, thus enhancing the performance of the proposed EADD-YOLO on the disease detection task.

## 3 Results

In this section, the experimental environment and hyperparameter settings are described detailed in subsection 3.1. In addition, the partitioning of the dataset is shown in subsection 3.1. Then, subsection 3.2 provides the evaluation metrics used in this study and their principles and calculation formulas. Finally, the experimental results are presented and analyzed in subsection 3.3 and subsection 3.4, including the lightweight of the backbone network and ablation studies.

### 3.1 Implementations and settings

The experiments are carried out on a 64-bit Windows operating system. The hardware configurations are as follows: Intel(R) Core (TM) i9-10900K CPU @ 3.70GHz, 64G memory, NVIDIA GeForce RTX3090. The version of PyTorch is 1.9.1.

In terms of training strategies, the hyperparameters are determined as follows: the number of epochs is 300, the batch size is 16, the initial learning rate is 1e-2, the weight decay is 0.0005, the momentum is 0.937, and the optimizer is SGD.

All the data are firstly divided into the initial training set and test set in the proportion of 8:2. Then, the initial training set is separated into the final training set and validation set for cross-validation in a ratio of 9:1 during training. The specific composition of the training set, validation set, and test set can be checked in the supplementary material.

### 3.2 Evaluation indicators

To evaluate the detection performance of the proposed EADD-YOLO, precision (P), recall (R), and mean average precision (mAP) are employed as evaluation indicators. Precision indicates the proportion of the number of positive samples correctly predicted to the number predicted as positive samples by the model. Recall denotes the proportion of correctly identified positive samples out of all positive samples. The former measures the precision of the network in identifying positive samples, and the latter represents the ability of the model to find positive samples. The mAP refers to the average value of the average precision of all classes, with higher values indicating higher detection accuracy of the model on the given dataset. They are calculated as follows:


(15) $$P=\frac{TP}{TP+FP}$$

(16) $$R=\frac{TP}{TP+FN}$$

(17) $$AP=\int_{0}^{1}P\left(R\right)\;dR$$

(18) $$mAP=\frac{\sum_{i=1}^{n}AP_{i}}{n}$$

where *TP* represents the number of positive samples that are correctly classified. *TN* is the number of negative examples that are correctly identified. *FP* denotes the number of negative examples that are misclassified as positive examples. *FN* indicates the number of positive samples that are incorrectly classified as negative examples.

Furthermore, parameter quantity and FLOPs are utilized to evaluate the size and computational cost of the model. The smaller number of parameters and FLOPs means less memory usage and faster computational efficiency of different networks. In addition, frames per second (FPS) is used to measure the detection speed of the model, i.e., the number of images that can be processed per second, with high values indicating faster inference speed.

### 3.3 Comparison of lightweight backbone

Before conducting the experiments, the performance of YOLOv5 with different sizes is compared on the ALDD test set, and the objective metric results are provided in the supplementary material. The results show that YOLOv5s has the advantage of high detection speed and small model size while maintaining good detection accuracy. As this study aims to propose a fast and easily deployable method for apple leaf disease detection with low loss of accuracy, YOLOv5s is selected as a benchmark model for subsequent experiments.

In this subsection, the backbone network of YOLOv5s is reconstructed by applying the basic module of several of the best-performing lightweight classification networks at the present stage. YOLO-GN represents that the backbone network of YOLOv5s is built utilizing the basic unit of GhostNet (Han et al., 2020). YOLO-ENL denotes applying the main module of EfficientNet-Lite (Tan and Le, 2019) to reconstruct the backbone network of YOLOv5s. YOLO-MN3s and YOLO-PPLCN indicate that their backbone networks are designed by employing the basic modules of MobileNetv3small (Howard et al., 2019) and PP-LCNet (Cui et al., 2021), respectively. The lightweight network using SNIR as the basic unit of the backbone network is denoted as YOLO-SNIR. The evaluation results of the YOLO model with different reconfigured backbone networks on the ALDD test set are displayed in **Table 2**.

**Table 2:** Comparison of experimental results of improved YOLOv5s with different lightweight backbones.

Model	P/%	R/%	mAP/%	Parameters/M	FLOPs/G	FPS
YOLOv5s	93.6	93.7	96.4	7.02	15.8	435
YOLO-GN	93.5	93.3	96.2	5.37	8.2	417
YOLO-ENL	93.2	91.6	95.5	3.78	7.2	455
YOLO-MN3s	92.2	91.5	95.2	3.54	6.1	588
YOLO-PPLCN	93.3	92.7	95.7	3.29	6.0	588
YOLO-SNIR	92.7	92.1	95.3	** 3.19**	** 5.9**	**625**

The bold font is to highlight the advantages of YOLO-SNIR in terms of the number of parameters, calculation, and inference speed.

As seen from **Table 2**, introducing the basic modules of different lightweight networks to construct backbone networks can reduce the number of parameters and FLOPs, despite causing the inevitable accuracy loss. Compared to YOLO-GN, YOLO-ENL, YOLO-MN3s and YOLO-PPLCN, YOLO-SNIR has a faster detection speed and smaller model size. In other words, the reconstruction of the backbone network of YOLOv5s employing the SNIR module has the most apparent enhancement in the network detection speed and compression of model size. Specifically, the mAP of YOLO-SNIR decreases by only 1.1% compared with YOLOv5s, while the parameter quantity and FLOPs of YOLO-SNIR are reduced by 54.56% and 62.66%, respectively. Moreover, the detection speed of YOLO-SNIR is 43.68% faster than that of YOLOv5s. In summary, introducing the efficient SNIR module to design the backbone of YOLOv5s is most appropriate for compressing the model size and improving detection speed.

### 3.4 Ablation studies

In this subsection, ablation experiments are conducted in two separate stages. In the first stage, the impact of lightweight improvements in network structure on detection performance is verified. In the second stage, the performance changes induced by methods of accuracy enhancement are demonstrated based on the lightweight model.

First, the impact of different lightweight improvement methods on the network performance is verified on the ALDD test set. Test 1 is the benchmark model YOLOv5s. Test 2 represents the reconstruction of the backbone network of the originalYOLOv5s employing the SNIR module. Test 3 denotes replacing the original C3 module with the proposed DWC3 module in the neck network of the original YOLOv5s. Lightweight-YOLO in Test 4 combines improvements from Tests 2 and 3. The evaluation results of the influence of the different improved structures on the network performance are displayed in **Table 3**.

**Table 3:** The results of different lightweight improvement methods on the YOLOv5s.

Test.No	Model	P/%	R/%	mAP/%	Parameters/M	FLOPs/G	FPS
1	Baseline (YOLOv5s)	93.6	94	96.4	7.02	15.8	435
2	YOLO-SNIR	92.7	92.1	95.3	3.19	5.9	625
3	YOLO-DWC3	93.6	92.4	96.0	6.00	13.7	454
4	Lightweight-YOLO	91.2	89.3	94.1	**1.96**	**3.6**	**667**

The bold font is to highlight the advantages of Lightweight-YOLO interms of the number of parameters, calculation, and inference speed.

As shown in **Table 3**, comparing Test 1 and Test 2, the mAP of YOLO-SNIR is 1.1% lower than that of YOLOv5s, but the detection speed is increased by 190 FPS. In addition, the number of parameters and FLOPs of YOLO-SNIR have decreased by 54.56% and 62.66% compared to YOLOv5s, respectively. The results illustrate that the backbone network composed of SNIR modules can significantly reduce the computational cost and improve the processing speed of the network with less influence on detection accuracy. From Tests 1 and 3, YOLO-DWC3 decreases the parameter quantity and FLOPs by 1.02 M and 2.1 G with only a 0.4% reduction in mAP, respectively. It indicates that introducing the proposed DWC3 module into the neck network not only compresses the model size but also almost maintains the detection performance of the network. The reason is that the novel DWC3 module allows for faster processing of input features by applying the proposed basic blocks while maintaining the ability of the network to understand them. From Test 4, compared with YOLOv5s, the detection accuracy of Lightweight-YOLO, which employs the SNIR module as the basic unit of the backbone network and introduces the proposed DWC3 module in the neck network, is decreased by 2.3%. The detection speed reaches 667 FPS, which is 1.53 times that of the original YOLOv5s. In addition, the parameter quantity and FLOPs of Lightweight-YOLO are only 27.92% and 22.78% of those for YOLOv5s, respectively. The results demonstrate the effectiveness of the proposed lightweight improvement methods.

From **Table 3**, it is clear that the lightweight of the network structure can significantly compress the model size and improve detection speed at the expense of reducing the accuracy of the network. Therefore, some improvement methods that can enhance accuracy without introducing high computational costs are essential.

Next, the changes in model performance caused by the introduction of the CA module and SIoU loss are verified using the Lightweight-YOLO as the base network. Test 4 is the Lightweight-YOLO with the introduction of SNIR and DWC3 modules. Test 5 introduces the CA module based on Test 4, while Test 6 replaces the original CIoU loss with SIoU loss based on Test 4. Test 7 is the proposed EADD-YOLO in this study, which represents introducing both CA and SIoU loss in Lightweight-YOLO. The impact of different improvement methods on the performance of Lightweight-YOLO is shown in **Table 4**, respectively. Moreover, the comparison of detection performance of different improved structures presented as Pareto frontier and the curves of changes in different metrics, including mAP, precision and recall during training, are provided in the supplementary material to further validate the results displayed in **Tables 3**, **4**.

**Table 4:** The results of introducing different improvements on the Lightweight-YOLO.

Test.No	Model	P/%	R/%	mAP/%	Parameters/M	FLOPs/G	FPS
4	Baseline (Lightweight-YOLO)	91.2	89.3	94.1	1.96	3.6	667
5	Lightweight-YOLO-CA	92.7	90.8	94.9	2.01	3.7	625
6	Lightweight-YOLO-SIoU	91.8	91.6	94.7	1.96	3.6	667
7	EADD-YOLO	** 92.8**	** 91.9**	** 95.5**	2.01	3.7	625

The bold font is to highlight the advantages of EADD-YOLO in terms of the detection accuracy.

As demonstrated in **Table 4**, Tests 4 and 5 indicate that the introduction of the CA module in Lightweight-YOLO can effectively enhance the detection accuracy of the network. The mAP is increased by 0.8%, and the detection speed of the network is only decreased by 42 FPS. The results illustrate that embedding CA modules can improve the accuracy of the model by highlighting information helpful for disease spot detection while suppressing useless information. Although this approach incurs some computational costs, it only slightly impacts the running speed of the network. The conclusion could be drawn from comparing Test 4 and Test 6 that replacing the location loss from CIoU loss with SIoU loss enhances the accuracy of the network by 0.6% in mAP. In addition, it does not affect the model size or the detection speed. Since the SIoU loss with introducing an angle cost can quickly pull the prediction box to the axis where the target box is located, reducing the wandering of the predicted box around the target box and improving the regression accuracy. From Tests 4 and 7, the detection accuracy of the proposed EADD-YOLO is 1.4% higher than that of the Lightweight-YOLO, while the number of parameters and FLOPs only increase by 0.05 M and 0.1 G, respectively. Compared with the original YOLOv5s (as shown in Test 1 in **Table 3**), the detection accuracy of the EADD-YOLO is only decreased by 0.9%, while the parameter quantity and FLOPs are reduced by 71.37 and 76.58%, respectively, and the detection speed is enhanced to 1.44 times.

In summary, the proposed EADD-YOLO, with its low number of parameters and FLOPs, significantly improves the speed of disease spot detection while having a negligible impact on detection accuracy. Therefore, the proposed EADD-YOLO is more suitable for deployment on resource-constrained mobile devices and with the detection performance required for practical applications.

**Table 5** shows the AP results of the single disease category of EADD-YOLO, Lightweight-YOLO and YOLOv5s on the ALDD test set. It can be observed that the single-type AP values of the proposed method are significantly higher than that of Lightweight-YOLO in all five types of disease images. Compared with YOLOv5s, EADD-YOLO has a better detection accuracy on rust images, and the detection accuracy on mosaic images is the same as that of YOLOv5s. For the other three disease images, the detection accuracy of the proposed method is just slightly lower than YOLOv5s. Overall, the proposed EADD-YOLO still performs satisfactorily in the detection task for multiple diseases compared with YOLOv5s. Therefore, it is verified that the proposed EADD-YOLO can maintain good detection accuracy while significantly compressing the model size and increasing the detection speed.

**Table 5:** AP results for the single-type of different models on the ALDD test set.

Category	YOLOv5s	Lightweight-YOLO	EADD-YOLO
Alternaria blotch	96.3	94.6	95.7
Brown spot	94.0	90.4	92.2
Grey spot	96.6	93.0	94.4
Mosaic	96.2	93.8	96.2
Rust	98.7	98.7	99.1

**Figure 7:**
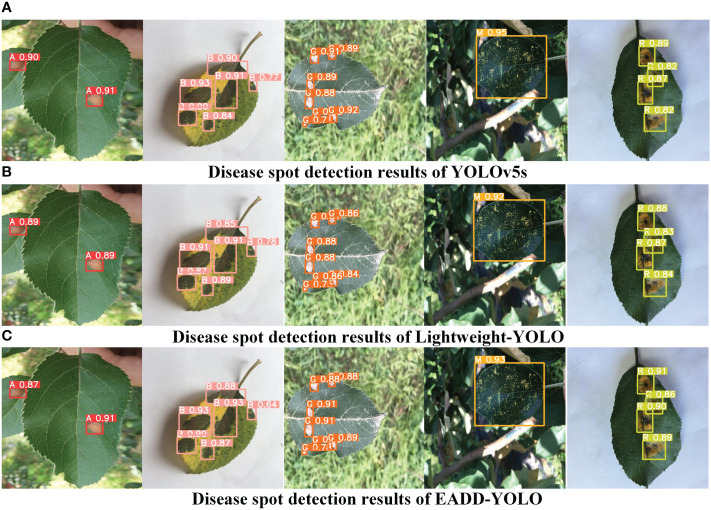
**(A-C)**Comparison of the detection effects of different models on five typical apple leaf disease images in simple scenes. The letters A, B, G, M, and R indicate Alternaria blotch, brown spot, grey spot, Mosaic, and rust, respectively.

**Figure 8:**
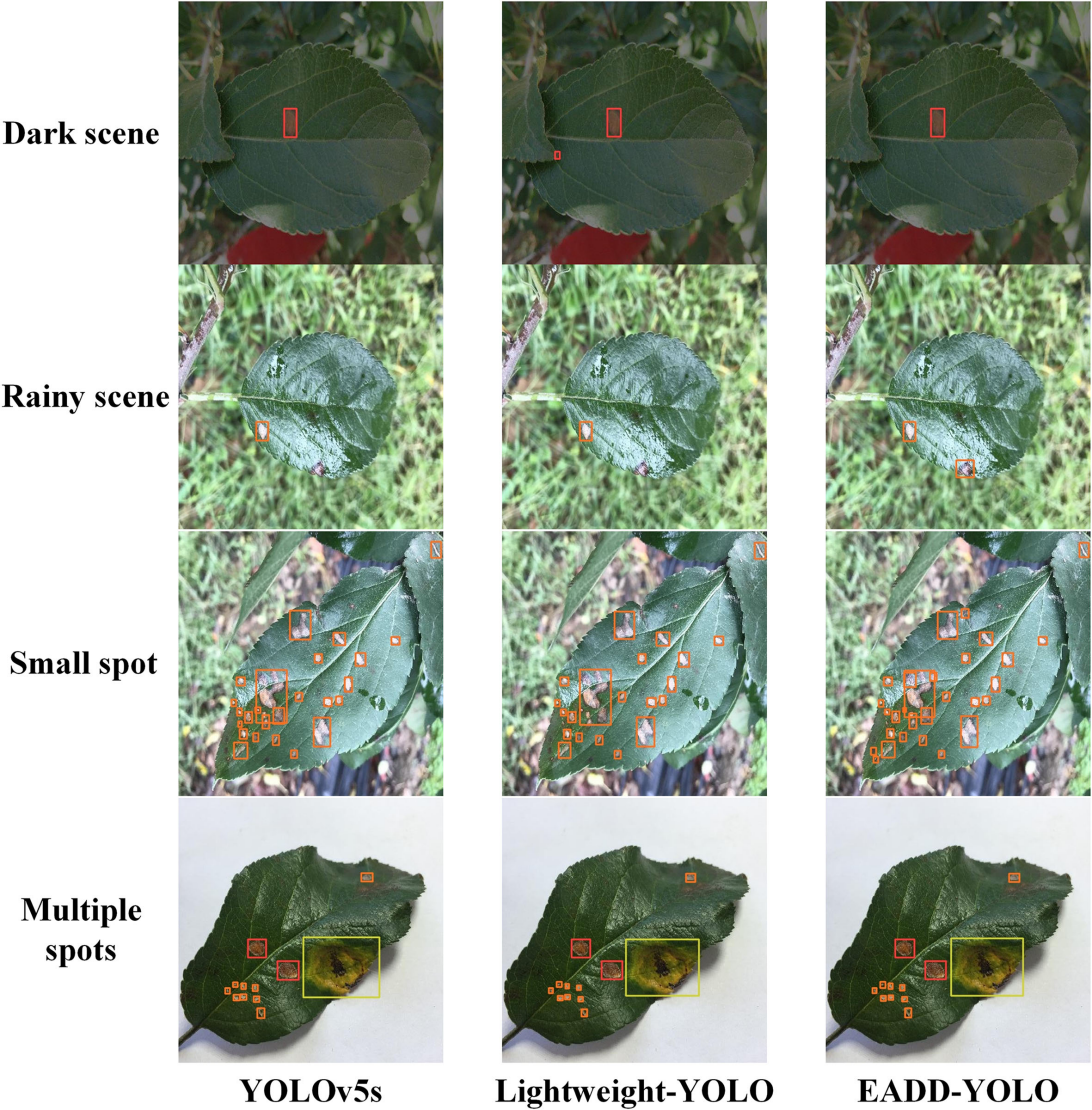
Comparison of the detection effects of different models on apple leaf disease images in special scenes.

To visualize the detection performance of the proposed method, the results of the disease spot images in different scenes are provided in **Figures 7** and **8**. More detection results are added in the supplementary material. **Figures 7A-C** display the detection results of YOLOv5s, Lightweight YOLO, and EADD-YOLO on five typical spot images in simple scenes, respectively. The Alternaria blotch, brown spot, grey spot, mosaic, and rust images are displayed from left to right. The predicted results in **Figure 7** are rendered to enhance readability by employing specific letters instead of disease-type names. It should be noted that the letter A indicates Alternaria blotch. The brown and grey spots are denoted by the letters B and G, respectively. In addition, the letters M and R represent mosaic and rust, respectively. As shown in **Figure 7**, for images in simple scenes and large spots, the detection performance of the proposed EADD-YOLO for different kinds of disease spots is higher than that of Lightweight-YOLO, and there is almost no difference compared with YOLOv5s. In particular, EADD-YOLO outperforms the original YOLOv5s in detecting rust. Because the proposed EADD-YOLO, which introduces the coordinate attention mechanism, has a better recognition ability for rust with distinct features.

To better demonstrate the superiority of the proposed method, **Figure 8** compares the detection performance of YOLOv5s, Lightweight-YOLO, and EADD-YOLO in special scenes. Because most of the spots are small and dense, labels and confidence levels are omitted to show the location of the predicted boxes more clearly. For the convenience of distinguishing, the disease spot categories represented by the prediction boxes of different colors are explained as follows: red indicates Alternaria blotch, pink denotes brown spot, orange and yellow represent grey spot and mosaic, respectively, and green refers to rust. From top to bottom are images of the dark light scene, the rainy scene, small spots, and images with multiple diseases.

As illustrated in **Figure 8**, for the disease image in the dark scene, Lightweight-YOLO mistakenly recognizes the dried area at the leaf tip as an Alternaria blotch, whereas EADD-YOLO does not. For the image from the rainy scene, EADD-YOLO can accurately detect the grey spot that is difficult to be identified due to rain reflection, but both YOLOv5s and Lightweight-YOLO miss the spot. Moreover, in the detection results for the image containing many dense and small spots, the clustered multiple diseases are recognized as one spot by YOLOv5s and Lightweight-YOLO, while EADD-YOLO can distinguish them separately. The results show that the lightweight of the network structure can undermine the detection effect, thus leading to the low detection performance of Lightweight-YOLO. In addition, EADD-YOLO, with the introduction of the coordinate attention mechanism, enhances the focus on spot information and thus performs better in detecting disease images in special scenes.

In summary, the detection results of the different models for disease images in various scenes in **Figures 7**, **8** are consistent with the pattern of objective indicators in **Table 5**. The comparison of the detection results demonstrates that EADD-YOLO offers excellent detection effects with low computational costs. Therefore, the proposed EADD-YOLO has better overall performance than the original YOLOv5s on the ALDD dataset.

## 4 Discussion

In this section, the proposed method is compared with the approaches used in previous studies on leaf spot detection to further verify its performance. Specifically, the INAR-SSD (Jiang et al., 2019) and MEAN-SSD (Sun et al., 2021) are apple leaf spot detection models based on the ALDD dataset. YOLOv4 is applied to detect apple leaf disease by Khan et al. (2022). MobileNetv2-YOLOv3 is a lightweight leaf disease detection network employed by Liu and Wang (2020). The disease detection method of Wang et al. (2022b) is an optimized lightweight YOLOv5 (OL-YOLOv5). EADD-YOLO is the efficient and accurate network for detecting apple leaf disease proposed in this study. The hyperparameters of different methods are set according to the original document. The objective evaluation results of different methods are shown in **Table 6**. In addition, the comparison of the detection performance of different methods in other forms is provided in the supplementary material.

**Table 6:** The results of different leaf spot detection methods on the ALDD test set.

	INAR-SSD [15]	MEAN-SSD [35]	YOLOv4 [16]	MobileNetv2-YOLOv3 [19]	OL-YOLOv5 [39]	EADD-YOLO (ours)
Alternaria blotch	75.56	78.82	81.79	81.7	**96.3**	95.7
Brown spot	79.70	82.94	82.49	73.7	**94.0**	92.2
Grey spot	76.50	81.35	83.25	85.8	**96.2**	94.4
Mosaic	70.63	78.78	78.43	94.2	96.1	**96.2**
Rust	91.59	93.71	95.15	96.9	98.8	**99.1**
mAP/%	78.80	83.12	84.5	86.4	**96.3**	95.5
Parameters/M	24.5	22.4	63.96	65.23	6.39	**2.01**
FLOPs/G	90.47	85.47	45.3	15.0	15.2	**3.7**
FPS	23	29	57	152	455	**625**

The bold font denotes which model has the best performance on a particular metric.

As displayed in **Table 6**, compared with other relevant popular methods, the proposed method has remarkable advantages in detection speed and model size with good detection accuracy. Specifically, compared with INAR-SSD and MEAN-SSD, the detection accuracy of EADD-YOLO is 16.7% and 12.3% higher, and the detection speed is 602 FPS and 596 FPS faster, respectively. In addition, the proposed method has only 0.08%, 0.04%, 0.09% and 0.04% of the number of parameters and FLOPs of INAR-SSD and MEAN-SSD, respectively. From the comparison of the results of YOLOv4, MobileNetv2-YOLOv3, and the proposed method, the detection accuracy of YOLOv4 and MobileNetv2-YOLOv3 is 11% and 9.1% lower than that of EADD-YOLO. Moreover, the detection speed of EADD-YOLO is 10.96 times and 4.11 times that of them, respectively. Compared with the OL-YOLOv5, the proposed method is 180 FPS faster in the detection speed, while the detection accuracy is only 0.8% lower. In addition, the parameter quantity and FLOPs of EADD-YOLO are only 31.46% and 24.34% of OL-YOLOv5, respectively. The results indicate that the proposed method has significant superiorities over other leaf disease detection methods in overall performance.

**Figure 9:**
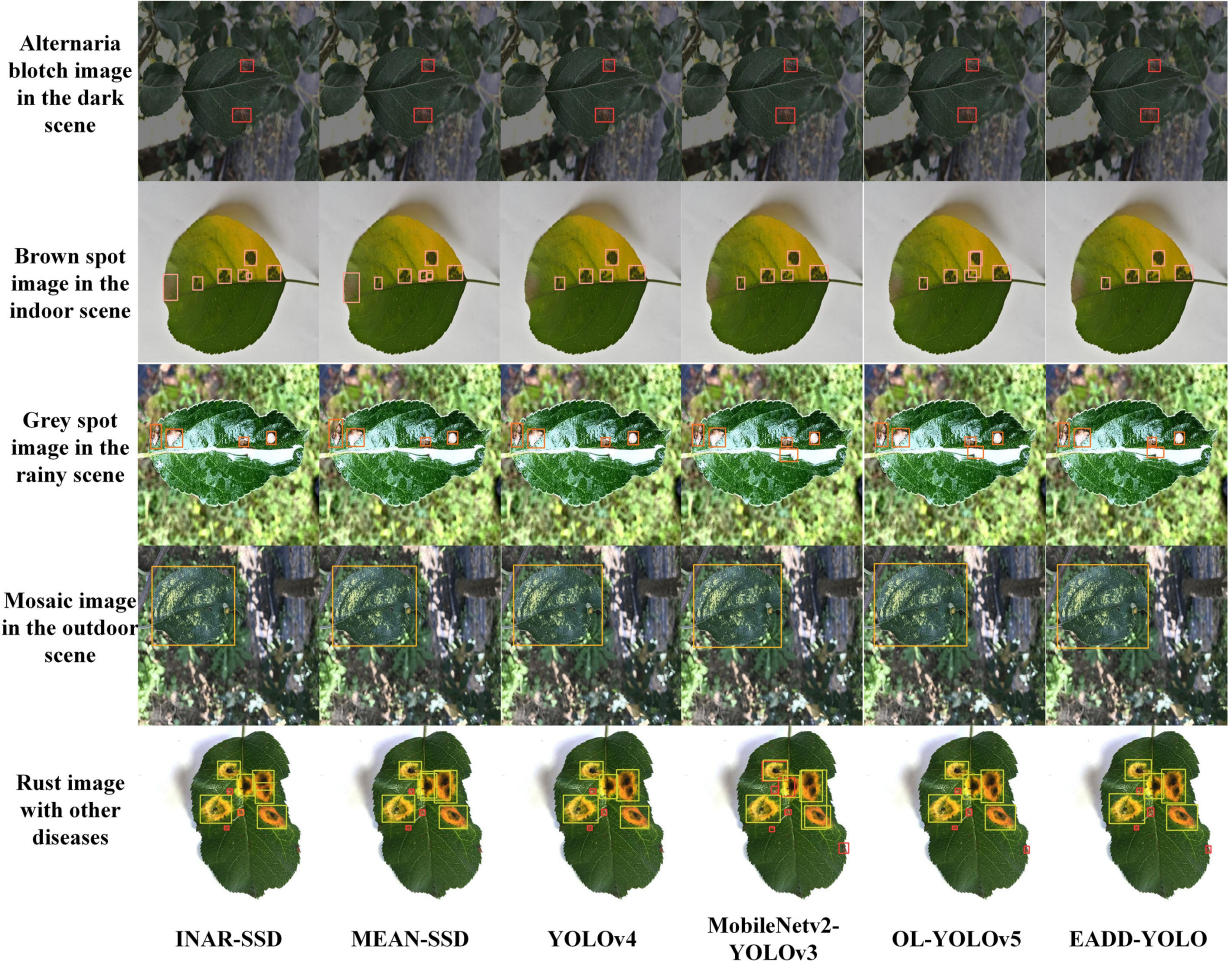
Comparison of the detection effects of different methods on five apple leaf disease images under different conditions.

**Figure 9** demonstrates the results of the different methods in detecting apple leaf spot images from various conditions, including the Alternaria blotch image in the dark scene, the brown spot image in the indoor background, the grey spot image in the rainy scene, the mosaic image in the outdoor environment and the rust image containing other diseases. Labels and confidence levels are omitted to show the location of the predicted boxes more clearly. The different colors of the prediction boxes indicate the different spot categories: red represents Alternaria blotch, pink denotes brown spot, orange indicates grey spot, and yellow and green represent mosaic and rust, respectively. More detection results of these methods are added in the supplementary material.

As displayed in **Figure 9**, the detection performance of EADD-YOLO on the diseased apple leaf images is better than INAR-SSD, MEAN-SSD, YOLOv4, and MobileNetv2-YOLOv3, while there is almost no difference from that of the OL-YOLOv5. Specifically, for the brown spot image in the indoor scene, both INAR-SSD and MEAN-SSD incorrectly regard the dust on the leaves as a grey spot, whereas the proposed EADD-YOLO does not. Moreover, INAR-SSD, MEAN-SSD and OL-YOLOv5 have the issue of repeated recognition of the brown spot area. EADD-YOLO can clearly identify every diseased area on the grey spot image from the rainy scene, while INAR-SSD, MEAN-SSD, and YOLOv4 omit the spot obscured by raindrops. For the rust image with other diseases, INAR-SSD, MEAN-SSD, and YOLOv4 ignore the small spot on the leaf edge. In addition, INAR-SSD, MEAN-SSD, and MobileNetv2-YOLOv3 have problems with inaccurate positioning of prediction boxes and duplicate rust spot identification. While the localization and recognition of the proposed EADD-YOLO for each disease area on the rust image containing multiple diseases are clear and accurate. The results illustrate that the ability of the proposed method to identify and locate the disease area is better than other leaf disease spot detection methods.

In summary, the proposed method shows satisfactory detection performance with minimal parameters and FLOPs. It can be concluded that the proposed method is superior to other popular methods in the task of leaf disease detection.

## 5 Conclusions

In this work, an efficient and accurate network for apple leaf spot detection, EADD-YOLO, is proposed to solve the problem of many parameters and the low efficiency of current disease detection algorithms. The backbone network of EADD-YOLO consists mainly of SNIR modules, which significantly reduces computational costs during feature extraction. The proposed DWC3 module is applied in the neck network to enhance the detection speed of the feature fusion. In addition, the introduction of the CA module and SIoU loss effectively compensates for the loss of accuracy caused by the lightweight design of the network. The experimental results show that the detection accuracy of EADD-YOLO is 95.5%, and the speed reaches 625 FPS. Compared with other methods, EADD-YOLO has a smaller model size and higher computational efficiency with excellent detection performance. Therefore, the proposed method provides technical support for the rapid diagnosis of early apple leaf diseases. In subsequent studies, the focus will be on further optimizing EADD-YOLO to extend it to more crop and fruit disease detection tasks.

## Data availability statement

The original contributions presented in the study are included in the article/**Supplementary Material**. Further inquiries can be directed to the corresponding author.

## Author contributions

JW and WM conceived and presented the main methodology. YW and CW contributed to the preparation of the equipment and the acquisition of the data. WM and SZ carried out the experiments and verified the methods. WM wrote the manuscript. JW and MY were responsible for checking and revising the manuscript. All authors contributed to the article and approved the submitted version.
