# Supplementary material for: EADD-YOLO: An efficient and accurate disease detector for apple leaf using improved lightweight YOLOv5
Source: Front Plant Sci. 2023 Feb 23;14:1120724. doi: 10.3389/fpls.2023.1120724 (PMC9996066; doi:10.3389/fpls.2023.1120724)
Supplement: Supplementary file 1 [file DataSheet_1.pdf]

Supplementary Material

1 SUPPLEMENTARY DATA

The representative images and their labels, and other supplementary tabels and figures are saved in a folder (Supplementary Material.zip, DOI: https://doi.org/10.6084/m9.figshare.21706868.v3).

2 SUPPLEMENTARY TABLES AND FIGURES

2.1 Tables

2.1.1 The specific composition and number of sub-datasets

Table S1 displays the specific composition and number of images of the training, validation, and test sets. It should be pointed out that the figures before and after the slash represent the number of indoor and outdoor images, respectively.

Table S1. The specific composition and number of sub-datasets.

	Alternaria blotch	Brown spot	Grey spot	Mosaic	Rust	Total
Training set	1264/2583	4072	1647/1816	2003/1507	3950/149	18991
Validation set	140/287	452	183/202	223/167	439/17	2110
Test set	351/718	1131	458/504	556/419	1097/42	5276
Total						26377

2.1.2 The performance of different sizes of YOLOv5

YOLOv5 includes four versions by setting different depth and width factors: YOLOv5s, YOLOv5m, YOLOv5l, and YOLOv5x. The performance of these models on the ALDD test set is shown in Table S2.

Table S2. Performance of different sizes of YOLOv5 on the ALDD test set.

Model	P/%	R/%	mAP/%	Parameters/M	FLOPs/G	FPS
YOLOv5s	93.6	93.7	96.4	7.02	15.8	435
YOLOv5m	93.6	94.0	96.7	20.87	48.0	238
YOLOv5l	93.7	94.0	96.8	46.13	107.9	185
YOLOv5x	93.9	94.2	97.0	86.20	204.1	110

The performance comparison in Table S2 illustrates that the YOLOv5s has the fastest processing speed while the YOLOv5x has the highest detection accuracy. Specifically, YOLOv5s has a detection accuracy of 96.4% and a detection speed of 435 FPS on the ALDD test set. The detection accuracy and speed of YOLOv5x are 97.0% and 110 FPS, respectively. The results demonstrate that the YOLOv5s is only 0.6% less than the YOLOv5x in terms of detection accuracy, while its speed is 3.95 times faster than YOLOv5x. In addition, the number of parameters and FLOPs of YOLOv5s are only 8.14% and 7.74% of those of YOLOv5x, respectively. So, it can be concluded that YOLOv5s has the advantage of high detection speed

and small model size while maintaining good detection accuracy. As this study aims to propose a fast and easily deployable method for apple leaf disease detection with low loss of accuracy, YOLOv5s is more suitable as a benchmark model for subsequent experiments.

2.2 Figures

2.2.1 Changes in several indicators for different improved models during training

Figure S1 displays the variation of several indicators of different improved models during the training process on the ALDD dataset, including mAP, precision and recall. It is evident that as the number of iterations increases, the metrics of each model grow rapidly and then become stable gradually. When the number of epochs reaches 300, the relationship between the detection performance of each model is consistent with objective indicators described in Tables 3 and 4 of the manuscript.

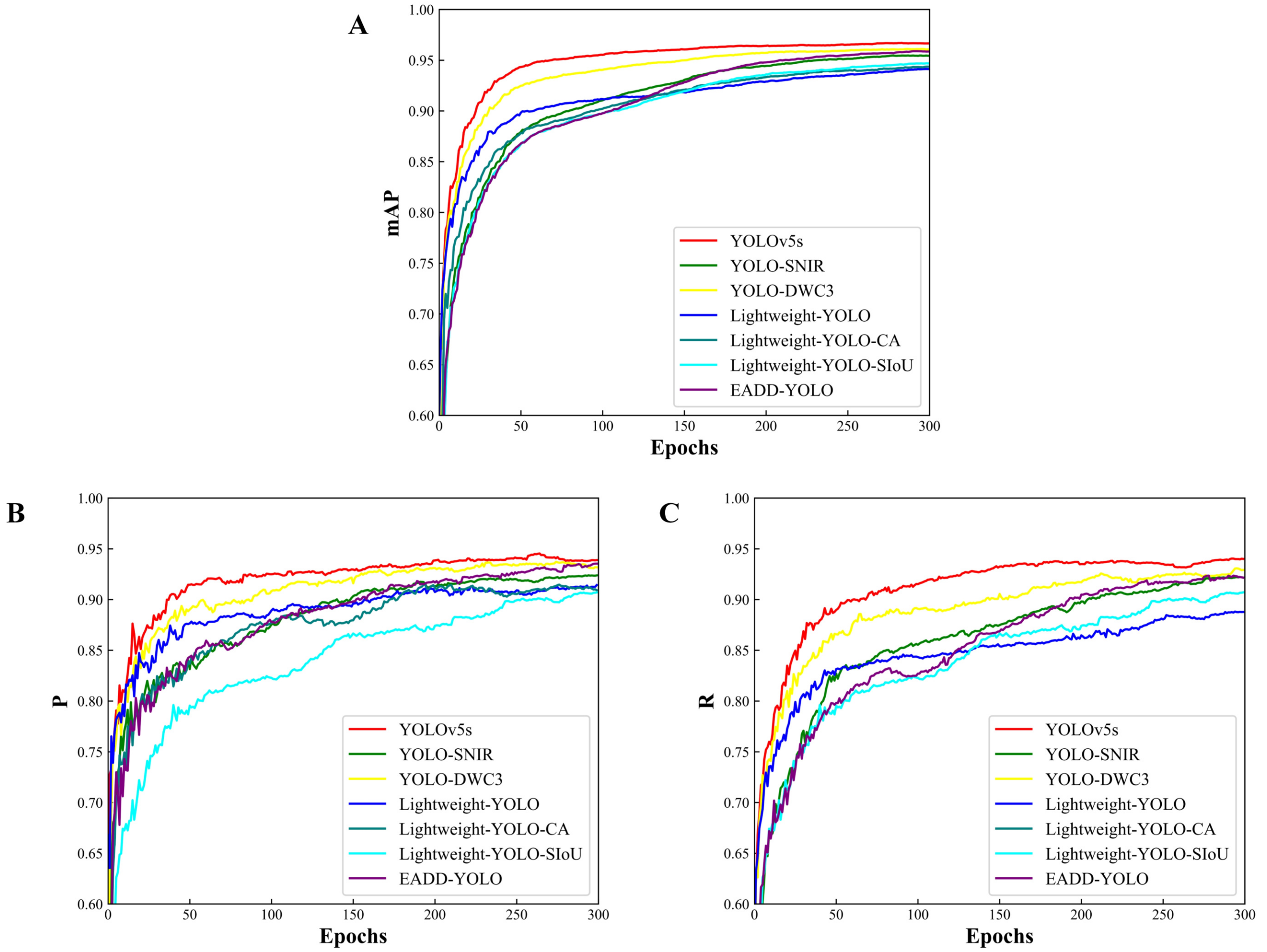

Figure S1. Variation of several indicators for different improved models during the training process. **(A)** The curve of changes in mAP. **(B)** The curve of changes in precision. **(C)** The curve of changes in recall.

2.2.2 Comparison of the performance of different improved models in the form of Pareto frontiers

Figure S2 demonstrates the performance differences between the different improved models in the form of Pareto frontiers. The blue bar chart describes the detection accuracy (mAP), while the grey, orange and yellow polylines illustrate the detection speed (FPS), parameter quantity (Params) and floating point of operations (FLOPs), respectively.

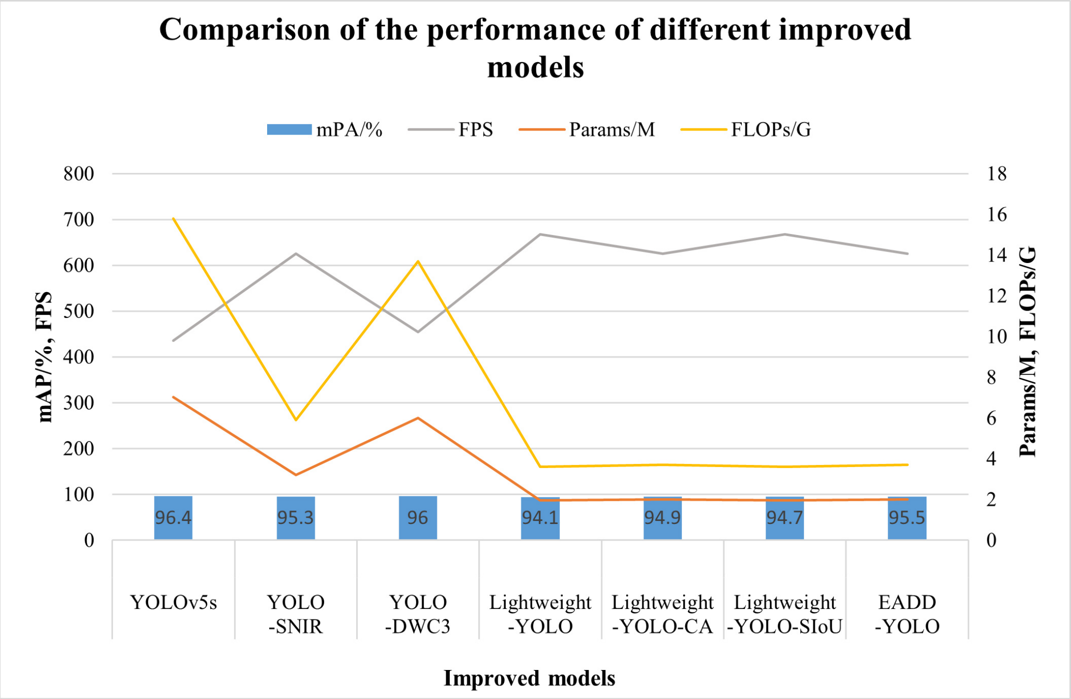

Figure S2. Comparison of the performance of different improved models.

As seen from Figure S2, the lightweight design, including improving the backbone network and introducing the proposed DWC3 module to the neck network, can significantly reduce the number of parameters and FLOPs with a slight loss in detection accuracy. In addition, introducing the CA module and SIoU loss enhances the detection accuracy of the network with little impact on the computational complexity. In the end, the proposed EADD-YOLO has smaller accuracy differences than the original YOLOv5s, but the number of parameters and FLOPs is significantly reduced, and the detection speed is also considerably increased. Therefore, EADD-YOLO has apparent advantages in terms of comprehensive performance.

2.2.3 The detection effects of different networks on the apple leaf disease images under various trained epochs

Figures S3, S4 and S5 display the detection results of YOLOv5s, Lightweight-YOLO and EADD-YOLO at different training periods. From left to right are the results for the different networks when trained for only 50, 100, 200 and 300 epochs, respectively.

As can be seen in Figures S3, S4 and S5, the overall detection effectiveness of the different networks for diseased leaf images improve as the number of training epochs increases. Of these, the detection

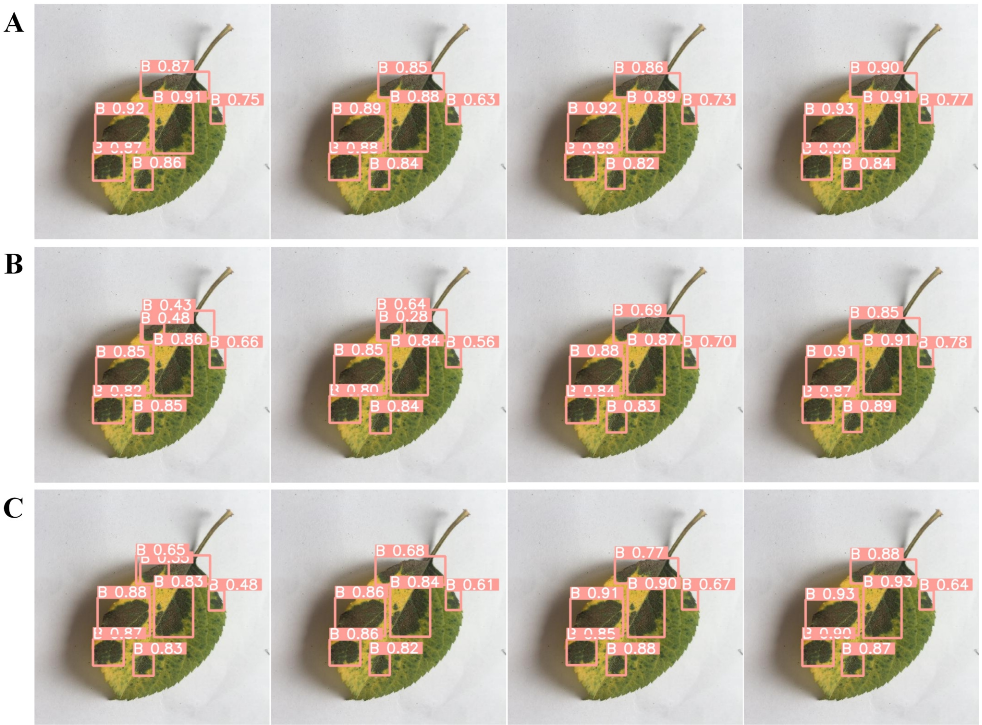

Figure S3. Comparison of the detection effects of different improved networks on the brown spot image under various trained epochs. **(A)** The detection results of YOLOv5s. **(B)** The detection results of Lightweight-YOLO. **(C)** The detection results of EADD-YOLO.

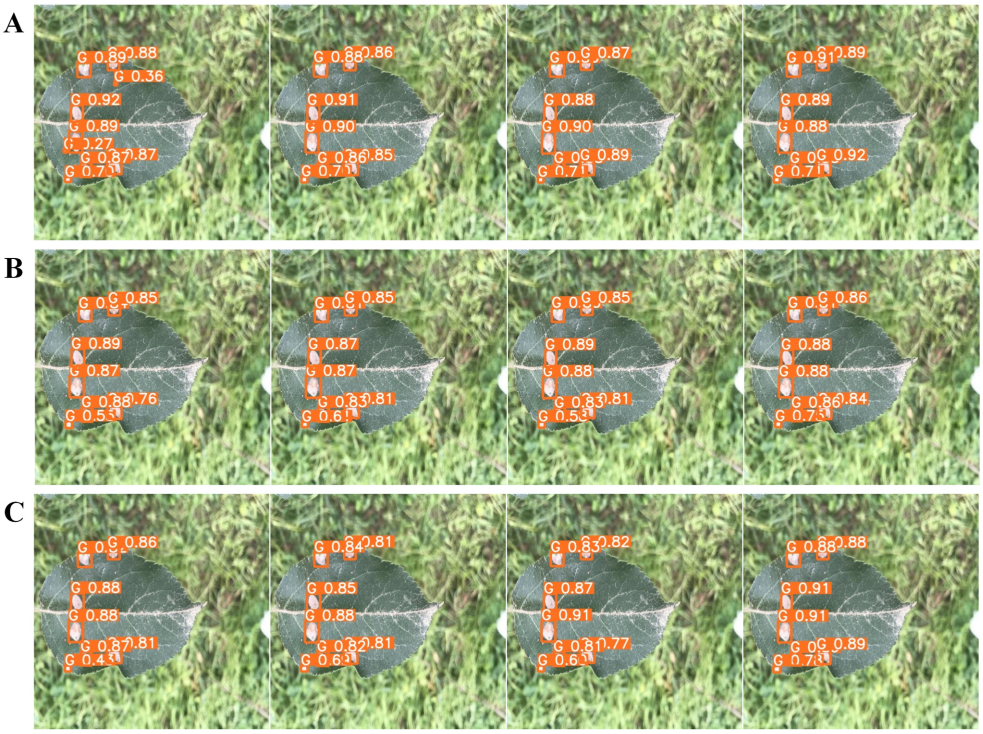

Figure S4. Comparison of the detection effects of different improved networks on the grey spot image under various trained epochs. **(A)** The detection results of YOLOv5s. **(B)** The detection results of Lightweight-YOLO. **(C)** The detection results of EADD-YOLO.

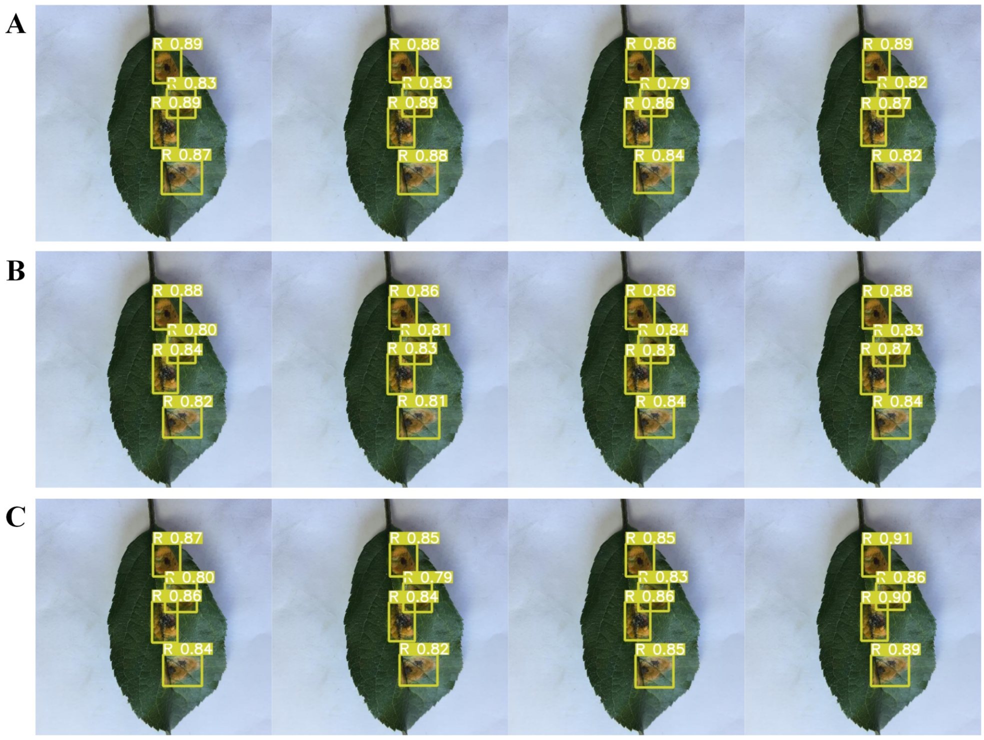

Figure S5. Comparison of the detection effects of different improved networks on the rust image under various trained epochs. **(A)** The detection results of YOLOv5s. **(B)** The detection results of Lightweight-YOLO. **(C)** The detection results of EADD-YOLO.

effectiveness of Lightweight-YOLO varies most significantly with training epochs, followed by EADD-YOLO's, with YOLOv5s showing the least significant change. Because of the simpler network structure of Lightweight-YOLO and EADD-YOLO, more extended training periods are required to fit the parameters. It is consistent with the pattern illustrated in Figure S3A for the change in mAP with epochs for different networks: the mAP for YOLOv5s plateaus at 100 training epochs, while Lightweight-YOLO and EADD-YOLO plateaus at 200 training epochs. To control the variables and ensure full integration of the models, we set the training period for all networks to 300 epochs. After 300 training epochs, it can be seen that EADD-YOLO outperforms Lightweight-YOLO in terms of overall performance and is not significantly different from YOLOv5s. In particular, EADD-YOLO outperforms the original YOLOv5s on the rust image, as demonstrated in Figure S3, because the introduction of the CA module improves the ability of the model to recognise rust diseases with distinct characteristics.

2.2.4 More detection results for different networks

To fully demonstrate the detection performance of EADD-YOLO, Lightweight-YOLO and YOLOv5s, more results of these models are displayed in Figure S6. Because most of the spots are small and dense, labels and confidence levels are omitted to show the location of the predicted boxes more clearly. For the convenience of distinguishing, the disease categories represented by the prediction boxes of different colors are explained as follows: red represents Alternaria blotch, pink denotes brown spot, orange and yellow indicate grey spot and mosaic, respectively, and green represents rust. From left to right are the Alternaria blotch image with environmental disturbances, the image of adhering brown spots, the small and dense grey spot image, the mosaic image in the dark scene and the rust image with other diseases.

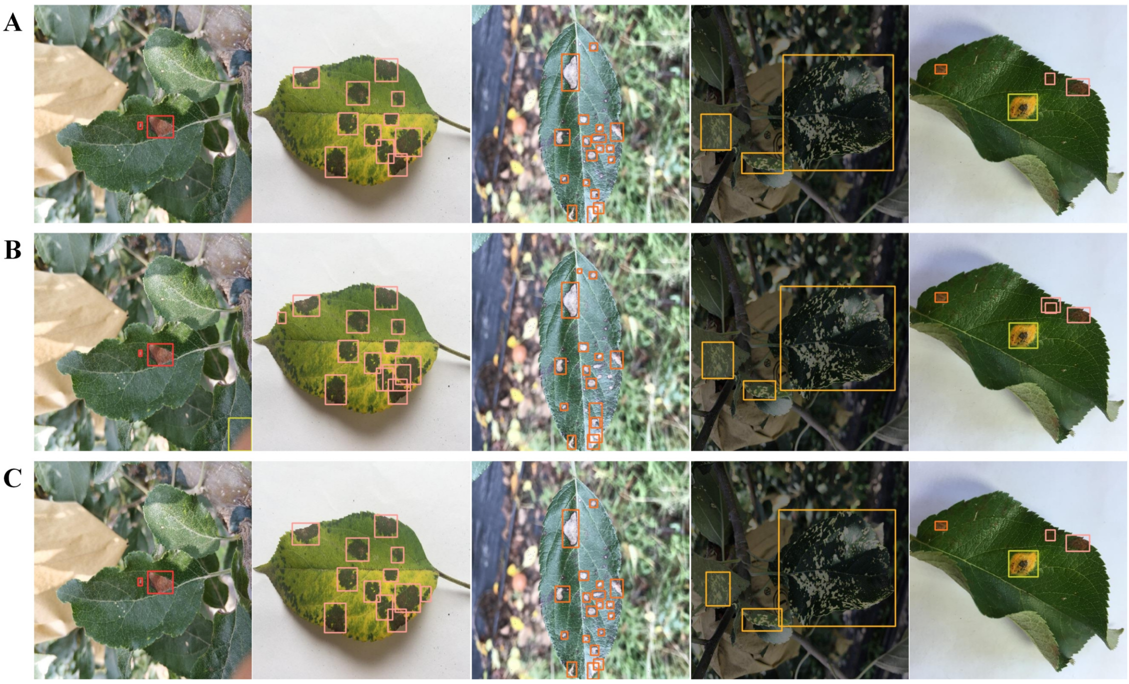

Figure S6. Comparison of the detection effects of different networks on apple leaf disease images with different conditions. **(A)** Disease spot detection results of YOLOv5s. **(B)** Disease spot detection results of Lightweight-YOLO. **(C)** Disease spot detection results of EADD-YOLO.

As shown in Figure S6, EADD-YOLO outperforms Lightweight-YOLO in detection, while there was little difference compared to YOLOv5s. Specifically, for the Alternaria blotch image, Lightweight-YOLO incorrectly identified elements in the lower right corner of the image that resembled the spot as a rust spot, while EADD-YOLO and YOLOv5s do not. Comparing the detection results of the brown spot image displays that both EADD-YOLO and YOLOv5s can identify and locate adherent spots more accurately than Lightweight-YOLO. For the small and dense grey spots, EADD-YOLO detects more small spots than the Lightweight-YOLO and YOLOv5s. The results from the mosaic and rust images illustrate that EADD-YOLO is more accurate in locating diseased areas than Lightweight-YOLO. In summary, the results demonstrate that the effective improvements introduced in EADD-YOLO compensate for the loss of accuracy caused by lightweight, thus giving it a similar performance to that of the original YOLOv5s.

2.2.5 Comparison of the performance of the proposed method with other relevant popular algorithms

To visualize the differences in detection performance between the different methods on the ALDD test set, Figure S7 demonstrates the bar charts comparing each method concerning detection accuracy, parameter quantity, FLOPs, and FPS. In addition, Figure S8 displays a comparison of the performance of the different methods in the form of Pareto frontiers. The blue bar chart describes the detection accuracy, while the grey, orange and yellow polylines illustrate the detection speed, parameter quantity and FLOPs, respectively. The results show that the proposed method maintains good detection accuracy with minimal computational costs and the fastest detection speed. It can be clearly seen that it is consistent with the results of the objective indicators in Table 6 of the manuscript.

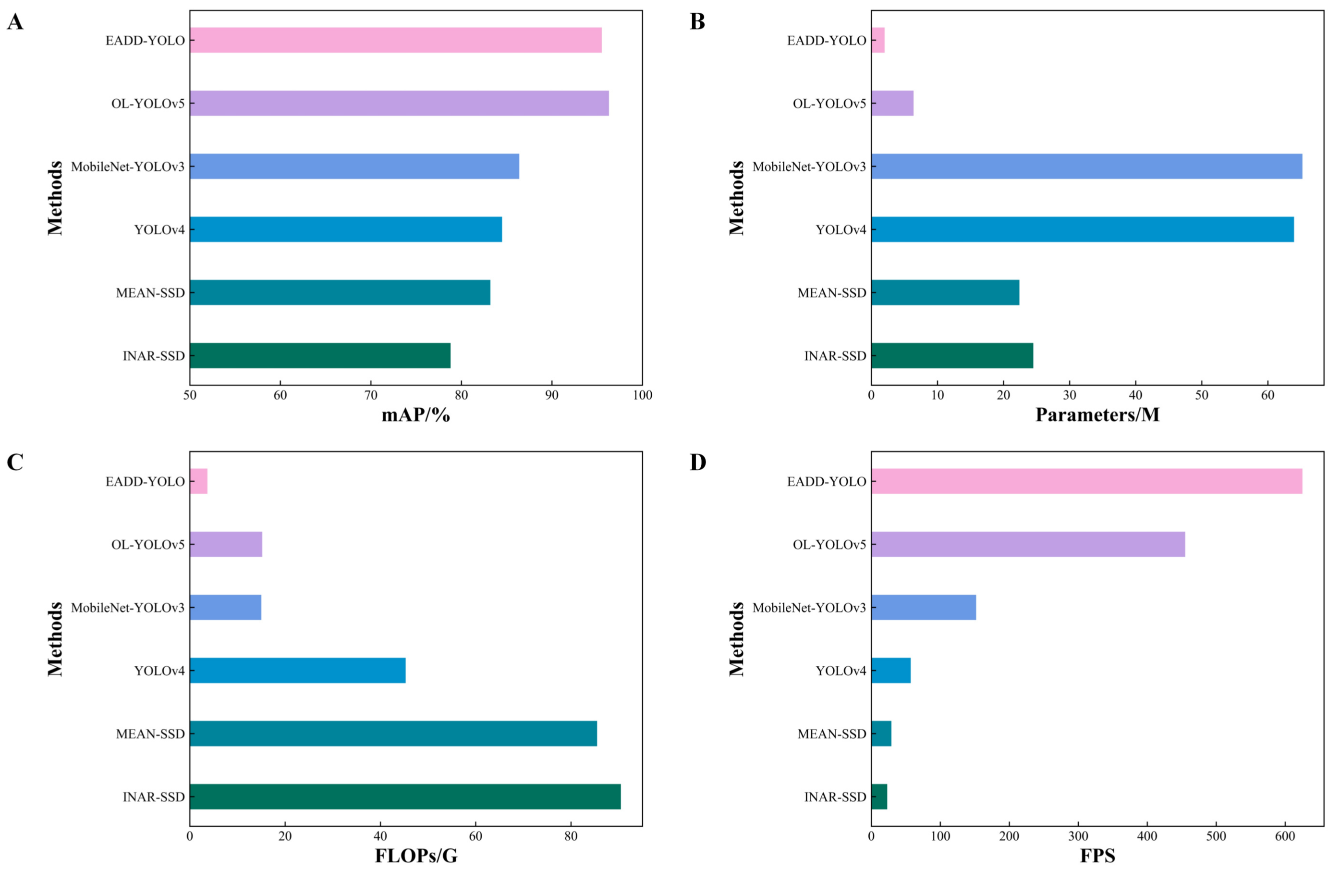

Figure S7. Comparison of the effectiveness of different methods on the ALDD test set. **(A)** Comparison of the different methods in mAP. **(B)** Comparison of the different methods in parameters. **(C)** Comparison of the different methods in FLOPs. **(D)** Comparison of the different methods in FPS.

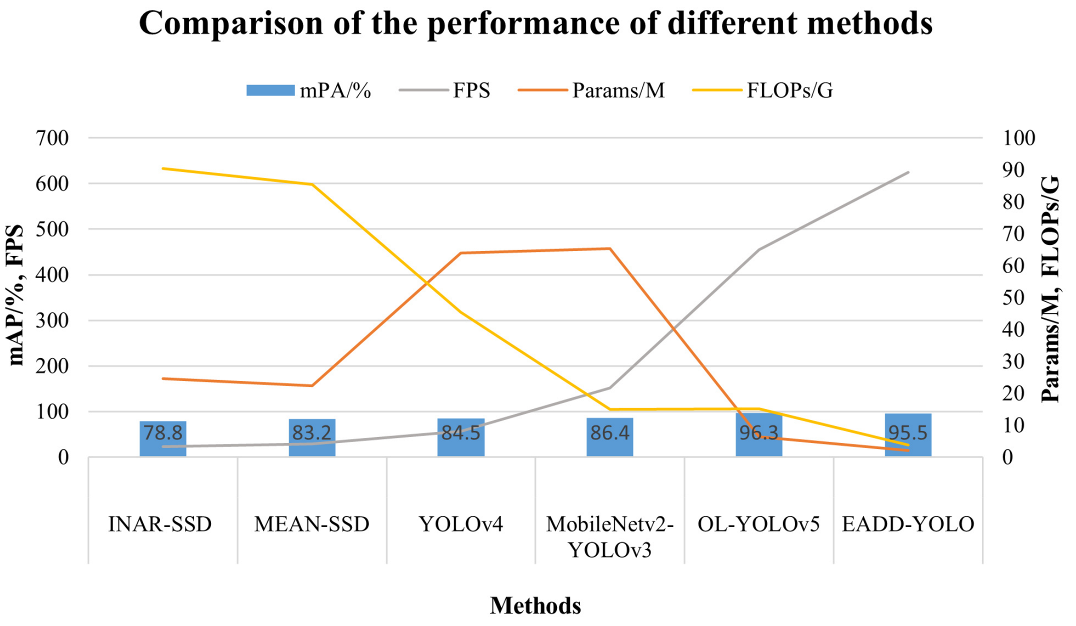

Figure S8. Comparison of the performance of different methods.

2.2.6 More results for different leaf disease detection methods

Figures S9, S10 and S11 display more detection results of the different methods in various conditions. In addition, labels and confidence levels are omitted to show the location of the predicted boxes more clearly. The different colors of the prediction boxes indicate the different spot categories: red represents Alternaria blotch, pink denotes brown spot, orange indicates grey spot, and yellow and green represent mosaic and rust, respectively.

The detection results in Figures S9, S10 and S11 illustrate that the detection performance of EADD-YOLO for apple leaf disease images is better than INAR-SSD, MEAN-SSD, YOLOv4, and MobileNetv2-YOLOv3, while there is almost no difference from that of the OL-YOLOv5. As shown in Figure S9, OL-YOLOv5 and the proposed EADD-YOLO can identify small spots on the Alternaria blotch and grey spot images that are missed by other methods. The phenomenon can also be found in the detection results of the Alternaria blotch and grey spot images in Figure S10. For the mosaic disease image with other diseases demonstrated in Figure S11, INAR-SSD, MEAN-SSD, and YOLOv4 ignore the small spots on the leaf edges, while EADD-YOLO can detect them. As seen from the detection results of brown spot images shown in Figures S9, S10 and S11, the proposed EADD-YOLO is more accurate in locating the diseased areas than other methods. From the detection results of the Alternaria blotch image in Figure S11, the proposed method can clearly identify the disease spots in the dark scene, while INAR-SSD, MEAN-SSD and YOLOv4 are affected by elements such as fingers and fruits in the background.

In summary, the proposed method shows satisfactory detection results with minimal parameters and FLOPs. It can be concluded that the proposed method is superior to other popular methods in leaf disease detection.

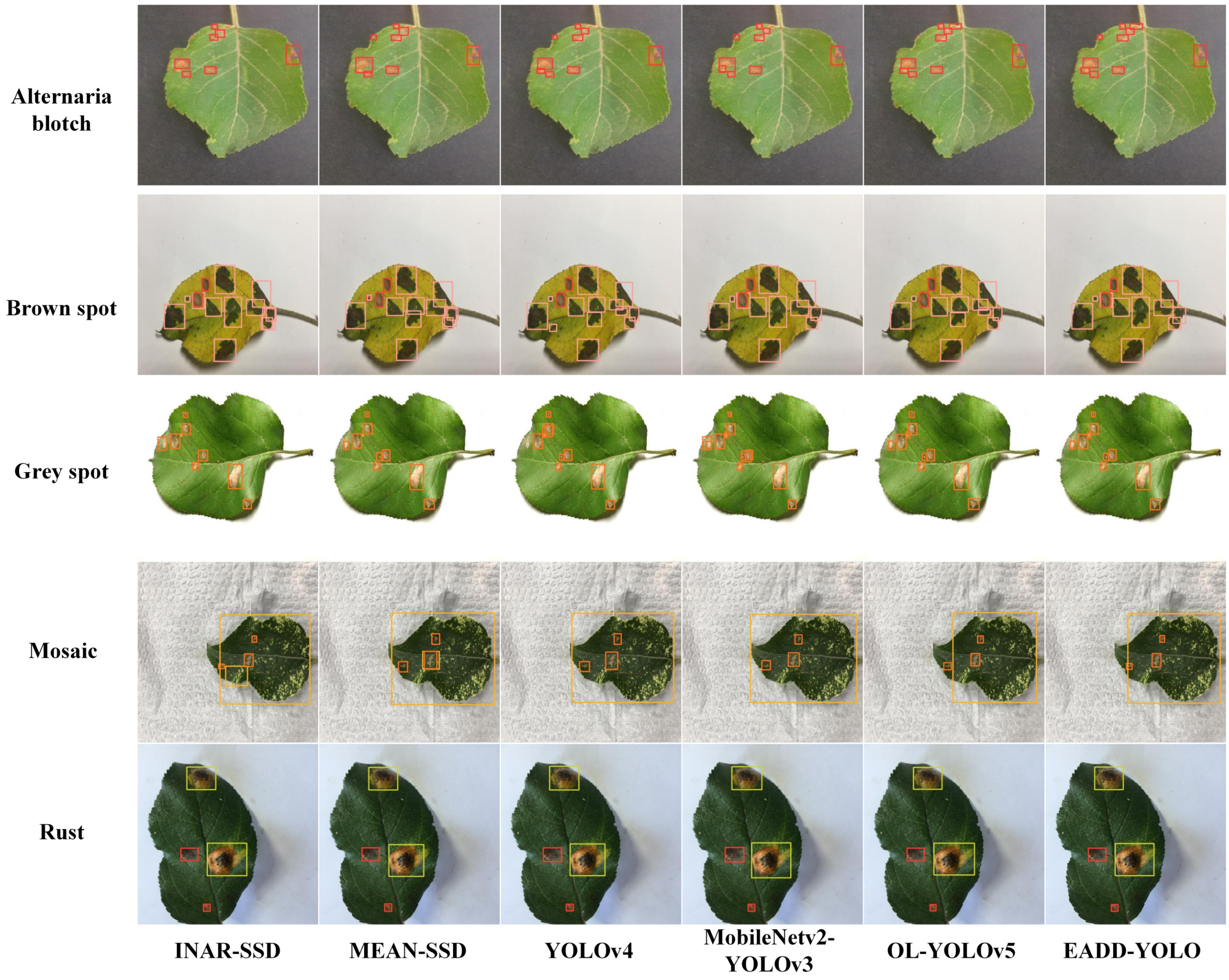

Figure S9. Comparison of the detection effects of different methods on five apple leaf spots under the indoor scene.

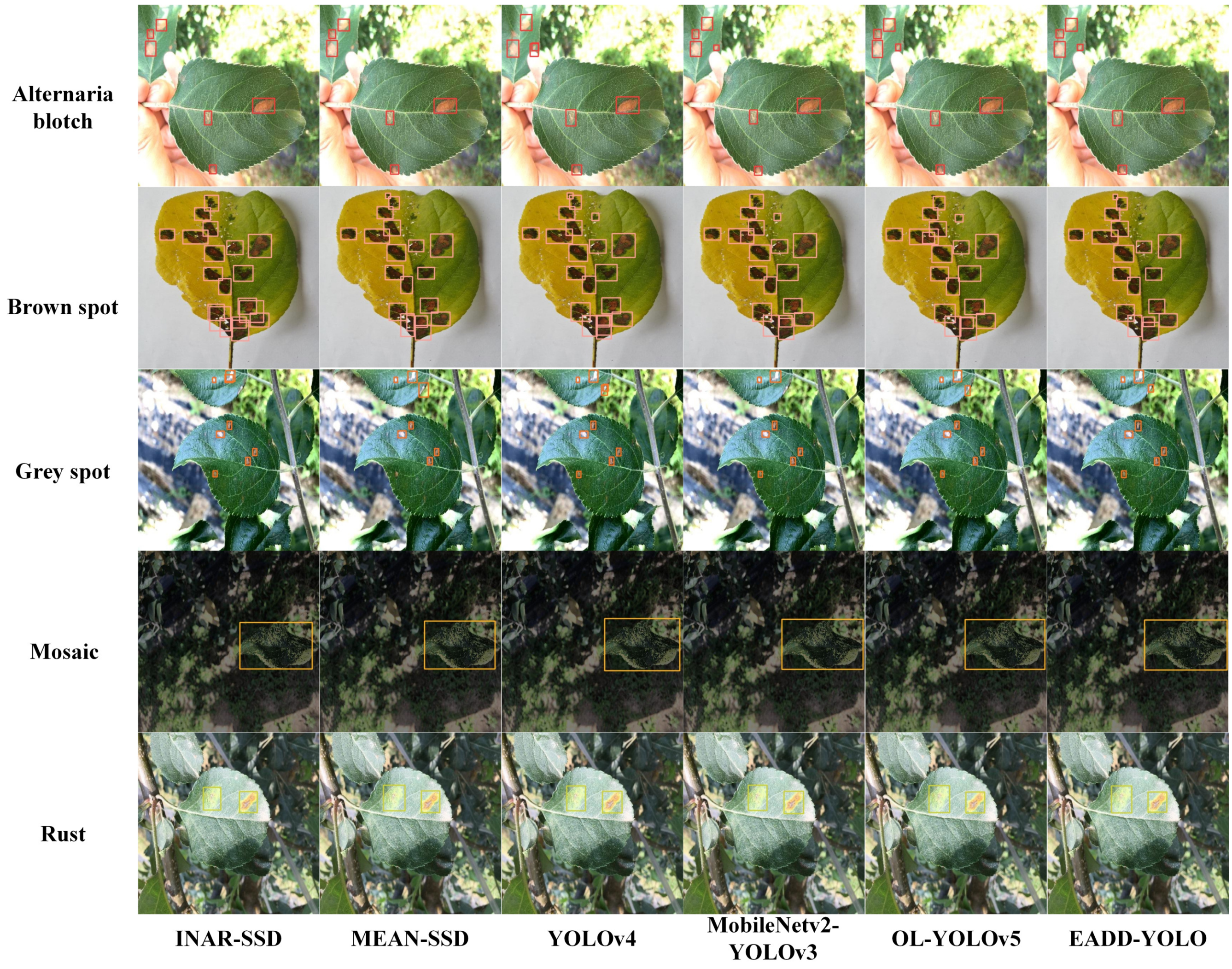

Figure S10. Comparison of the detection effects of different methods on five apple leaf spots under the outdoor scene.

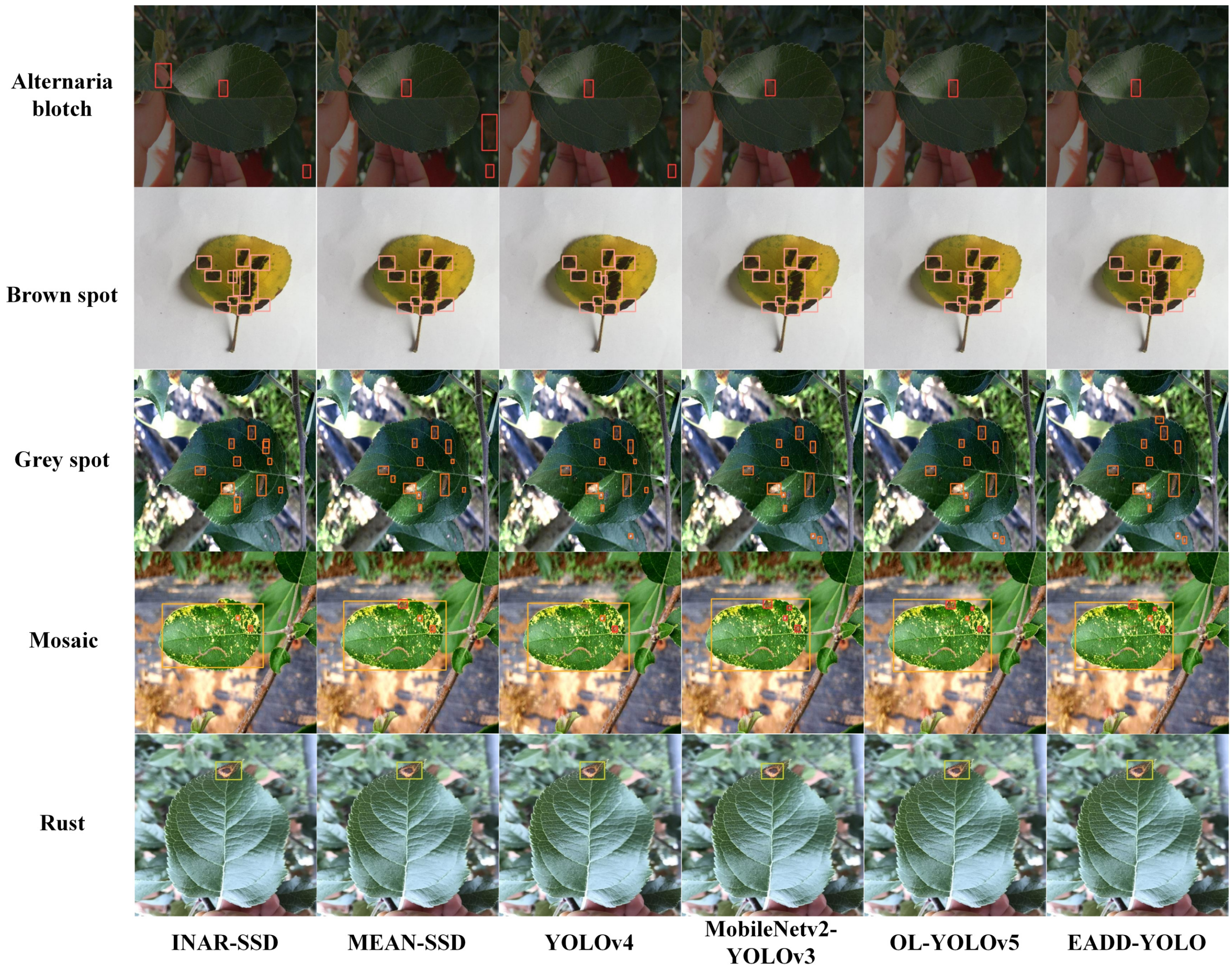

Figure S11. Comparison of the detection effects of different methods on five apple leaf spots under different conditions.
